# The role of conventional and unconventional adaptive routes in lowering of intraocular pressure: Theoretical model and simulation

**DOI:** 10.1063/5.0151091

**Published:** 2023-06-07

**Authors:** Riccardo Sacco, Greta Chiaravalli, Gal Antman, Giovanna Guidoboni, Alice Verticchio, Brent Siesky, Alon Harris

**Affiliations:** 1 Dipartimento di Matematica, Politecnico di Milano, 20133 Milano, Italy; 2 Center for Nanoscience and Technology, Istituto Italiano di Tecnologia, 20133 Milano, Italy; 3 Dipartimento di Fisica, Politecnico di Milano, 20133 Milano, Italy; 4 Department of Ophthalmology, Icahn School of Medicine at Mount Sinai, New York, New York 10029, USA; 5 Department of Ophthalmology, Rabin Medical Center, Petah Tikva 4941492, Israel; 6 College of Engineering, University of Maine, Orono, Maine 04469, USA

## Abstract

In this article, we propose a theoretical model leveraging the analogy between fluid and electric variables to investigate the relation among aqueous humor (AH) circulation and drainage and intraocular pressure (IOP), the principal established risk factor of severe neuropathologies of the optic nerve such as glaucoma. IOP is the steady-state result of the balance among AH secretion (AHs), circulation (AHc), and drainage (AHd). AHs are modeled as a given volumetric flow rate electrically corresponding to an input current source. AHc is modeled by the series of two linear hydraulic conductances (HCs) representing the posterior and anterior chambers. AHd is modeled by the parallel of three HCs: a linear HC for the conventional adaptive route (ConvAR), a nonlinear HC for the hydraulic component of the unconventional adaptive route (UncAR), and a nonlinear HC for the drug-dependent component of the UncAR. The proposed model is implemented in a computational virtual laboratory to study the value attained by the IOP under physiological and pathological conditions. Simulation results (*i*) confirm the conjecture that the UncAR acts as a relief valve under pathological conditions, (*ii*) indicate that the drug-dependent AR is the major opponent to IOP increase in the case of elevated trabecular meshwork resistance, and (*iii*) support the use of the model as a quantitative tool to complement *in vivo* studies and help design and optimize medications for ocular diseases.

## I. INTRODUCTION

In this article, we propose a theoretical model to investigate the processes of aqueous humor (AH) circulation and drainage in the human eye^1–4^ under the assumption of a given volumetric flow rate (VFR) of AH secreted by the ciliary processes.

The main clinical motivation of this study is that AH establishes the steady-state value of the intraocular pressure (IOP) in the globe of the eye.^5^ Lowering of IOP is the only currently approved therapeutic approach for a severe eye disease such as glaucoma, so that, being able to keep IOP at a controlled, healthy level, typically around 15 mm Hg, may prevent retinal ganglion cells and optic nerve head from irreparable damage.^6,7^ A pressure level of 21 mm Hg is usually taken as a measure for screening or diagnosis of glaucoma, even if several population-based studies indicate a great interindividual variation in the susceptibility of the optic nerve to IOP-related damage.^8^

An abnormal value of IOP is the macroscopic result of multiple disorders localized at very different spatial scales, ranging from $1\;\text{nm}=10^{-9}\;m$ (level of cellular plasma membrane of the ciliary epithelium) to $1\;\text{mm}=10^{-3}\;m$ (level of the trabecular meshwork, TM). To deal with such a wide spatial range in a computationally effective manner, in the present work, we develop a mathematical model of AH dynamics based on the average of the laws of mass and linear momentum balance of the aqueous humor fluid over the posterior chamber (PC), the anterior chamber, the trabecular meshwork, and the ciliary muscle. The result of the average is a zero-dimensional (0D, or compartment) representation of the anterior segment of the eye that includes

• a compartment representation of AH circulation from the posterior chamber (PC) into the anterior chamber (AC);
• a compartment representation of AH drainage through trabecular and uveoscleral (US) outflow pathways.

The advantage of averaging mass and momentum balance laws over the various regions of the anterior segment of the eye is that the resulting compartment representation can be conveniently described in mathematical terms by leveraging the analogy between fluid and electric variables illustrated in Ref. 9. AH circulation is the result of passive fluid diffusion driven by hydraulic pressure gradients across the series of two linear hydraulic conductances representing the posterior and anterior chamber.^10^ AH drainage is the result of adaptive mechanisms modeled by the parallel of two main outflow pathways, a “conventional” adaptive route (ConvAR) throughout the TM^11,12^ and an “unconventional” adaptive route (UncAR) throughout the ciliary muscle.^13^ A significant novelty of the formulation proposed in this article compared to the existing literature is the mathematical treatment of the UncAR. Based on the studies of Johnson and Erickson,^14^ Johnson *et al.*,^15^ and Costagliola *et al.*,^13^ the UncAR is modeled by the parallel of a pressure-dependent route and a drug-dependent route. The former is described by a nonlinear hydraulic conductance depending on the pressure difference between the AC and the suprachoroidal (Sch) space,^16^ the latter by a current source nonlinearly controlled by the amount of topical drug administered to lower the IOP.

The model is numerically implemented in a computational virtual laboratory (CVL) for subsequent use in the study of problems of clinical interest. In particular, the focus is devoted to determine the value attained by IOP under physiological and pathological conditions. In the former case, model parameters are calibrated to reproduce the characteristic values of AH volumetric flow rate and IOP in a normal healthy eye.^3^ In the latter case, pathological conditions are simulated by increasing the value of the TM resistance with respect to baseline conditions.^17,18^ Results from extensive simulations with the CVL:

(1) confirm the conjecture of Costagliola *et al.*^13^ that the UncAR acts as a relief valve under pathological conditions related to an abnormal increase of TM resistance;
(2) indicate that the drug-dependent AR is the major opponent to IOP increase due to elevated TM resistance;
(3) support the use of the model as a quantitative tool to complement in vivo studies and help design and optimize medications for ocular diseases.

An outline of the article is as follows. Section II gives an introduction to the physiological processes of AH dynamics in the human eye. Section III introduces the three-dimensional (3D) geometric and differential model to study AH circulation and drainage in the human eye. Section IV illustrates the procedure to reduce the 3D geometric and differential model to a compartment model based on a suitable average of the mass and momentum balance equation over each region of the anterior segment of the eye. In Sec. V, the reduced model is translated into a conceptual model by leveraging the analogy between fluid and electric variables, while in Sec. VI, the conceptual model is translated into a system of algebraic equations expressing Kirchhoff's law for the current at each node of the circuit and Ohm's law for the linear and nonlinear hydraulic conductances. Section VII describes the numerical values of model parameters that are used in the numerical simulations, and Sec. VIII describes the algorithm, based on a fixed-point iteration, to compute the intraocular pressure as a function of the other input variables and parameters. Section IX contains a detailed report of the computational tests that are performed to validate the proposed model and fixed-iteration algorithm in the study of two clinically relevant situations. Section X discusses the physiological consistency of the simulation results illustrated in Sec. IX and the clinical relevance of the adaptive routes of the unconventional outflow pathway on the lowering of IOP. Section XI summarizes the main findings of the article, indicating some limitations of the proposed model and suggestions to extend its use as a flexible tool to complement clinical practice and experimental activity. Section XII collects the figures illustrating the simulation results described in Sec. IX and discussed in Sec. X. Appendixes A and B illustrate the main results of the theoretical analysis of the solution map, providing sufficient conditions to be satisfied by model parameters to ensure the existence and uniqueness of the solution and the convergence of the fixed-point iteration.

## II. PHYSIOLOGY OF AQUEOUS HUMOR DYNAMICS

**FIG. 1.**
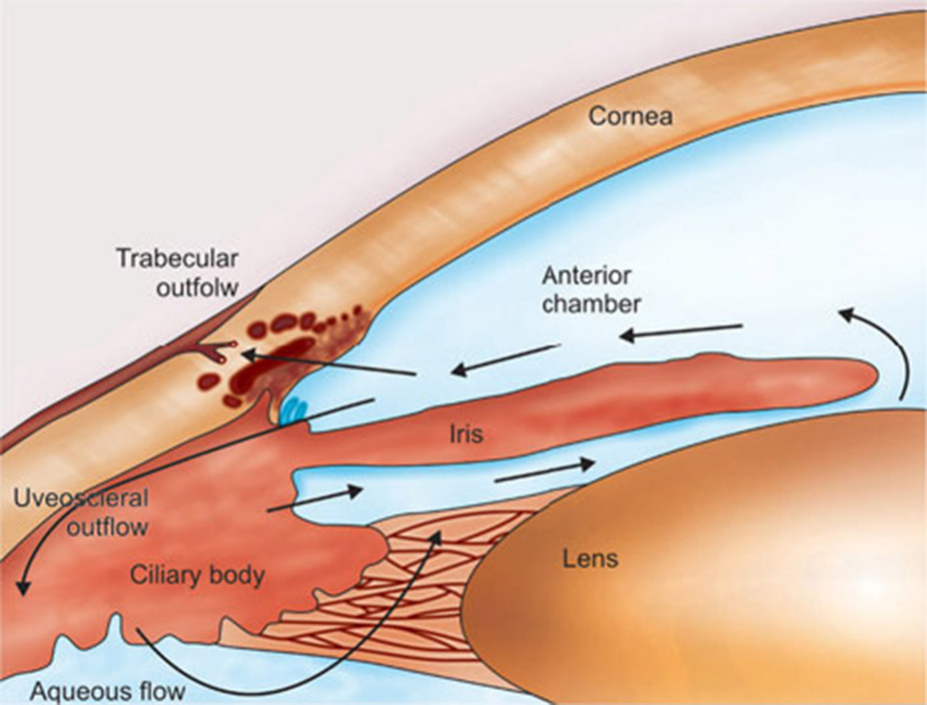
Inflow and outflow pathways of AH. Black arrows are used to indicate the secretion of AH from the ciliary body, its circulation from the PC, which is the region behind the iris and in front of the ciliary body and the lens, into the AC, and its drainage through the trabecular and uveoscleral outflow pathways. [Reprinted with permission from Ramakrishnan *et al.*, *Diagnosis and Management of Glaucoma*, Aqueous Humor Dynamics (Jaypee Brothers Medical Publishers 2013), Chap. 09. Copyright 2013 Authors, licensed under a Creative Commons Attribution (CC BY) license.]

**FIG. 2.**
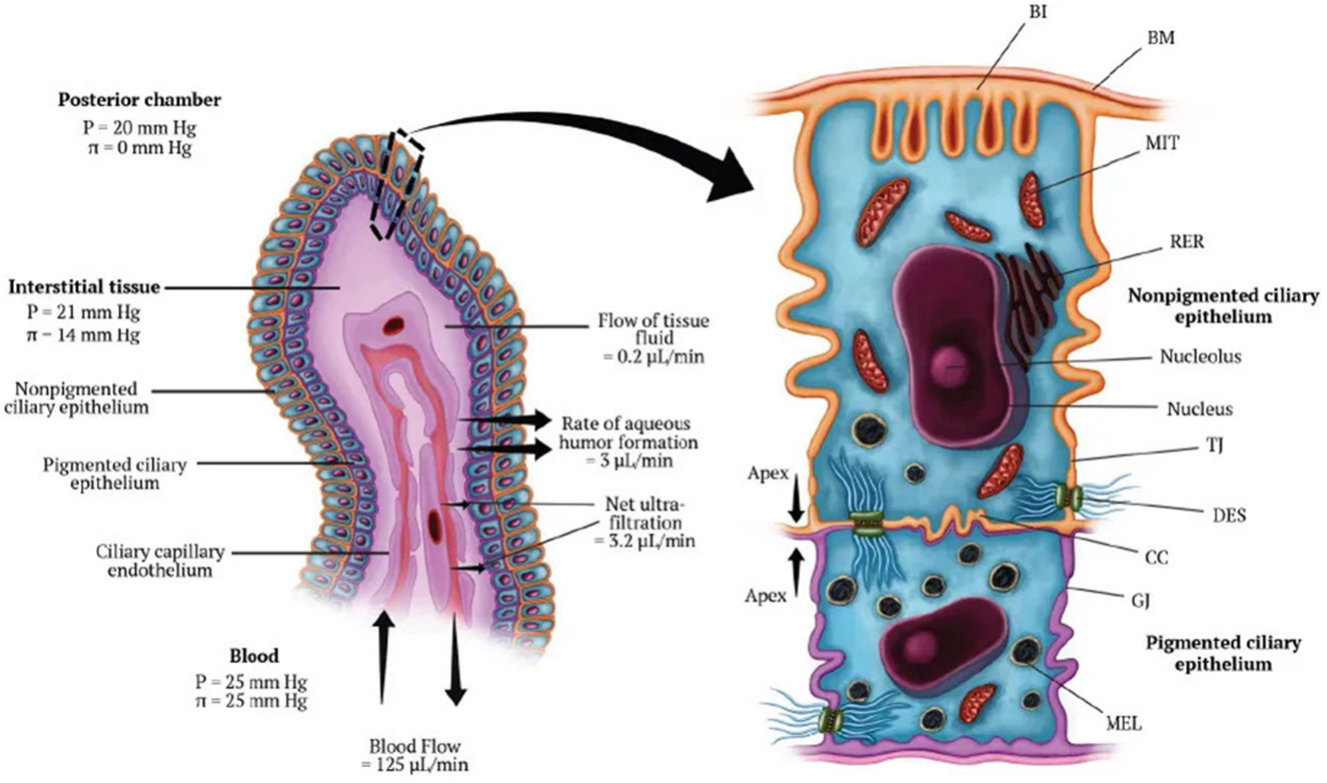
Ciliary processes and ciliary epithelium. [Reprinted with permission from S. Sampathkumar and C. B. Toris, “Minimally invasive glaucoma surgery. A practical guide,” in *IC Aqueous Production*, edited by B. A. Francis, S. R. Sarkisian, and J. C. Tan (Georg Thieme Verlag KG, Stuttgart, 2018, Chap. 13. Copyright 2018 Authors, licensed under a Creative Commons Attribution (CC BY) license.]

**FIG. 3.**
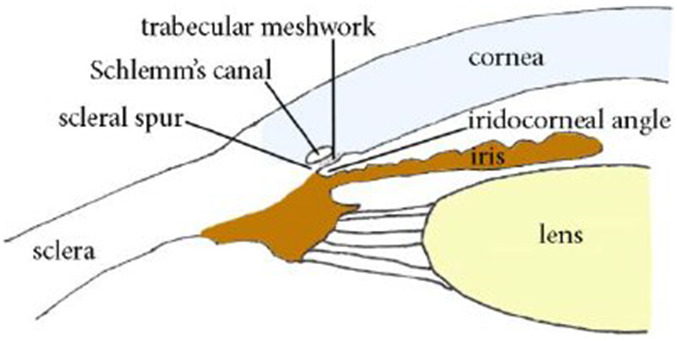
Cross-sectional view of the anterior segment of the eye highlighting the iridocorneal angle. [Reprinted with permission from Campa *et al.*, “Anterior chamber angle assessment techniques,” in *Glaucoma–Basic and Clinical Concepts*, edited by S. Rumelt (IntechOpen, 2011). Copyright 2011 Authors, licensed under a Creative Commons Attribution (CC BY) license.]

Figure 1 illustrates the processes contributing to AH dynamics, whereas Fig. 2 illustrates the structure of the ciliary epithelium. Aqueous humor is secreted by the ciliary epithelium into the PC, which is the region behind the iris and in front of the ciliary body and the lens. Then, the AH fluid is not stagnant, rather, it undergoes the following physical processes that regulate its motion throughout the anterior segment of the eye:

**M.1:** AH circulation from the PC, through the pupil, to the iridocorneal angle of the AC, shown in Fig. 3;
**M.2:** AH drainage through the TM outflow pathway and the US outflow pathway.

**FIG. 4.**
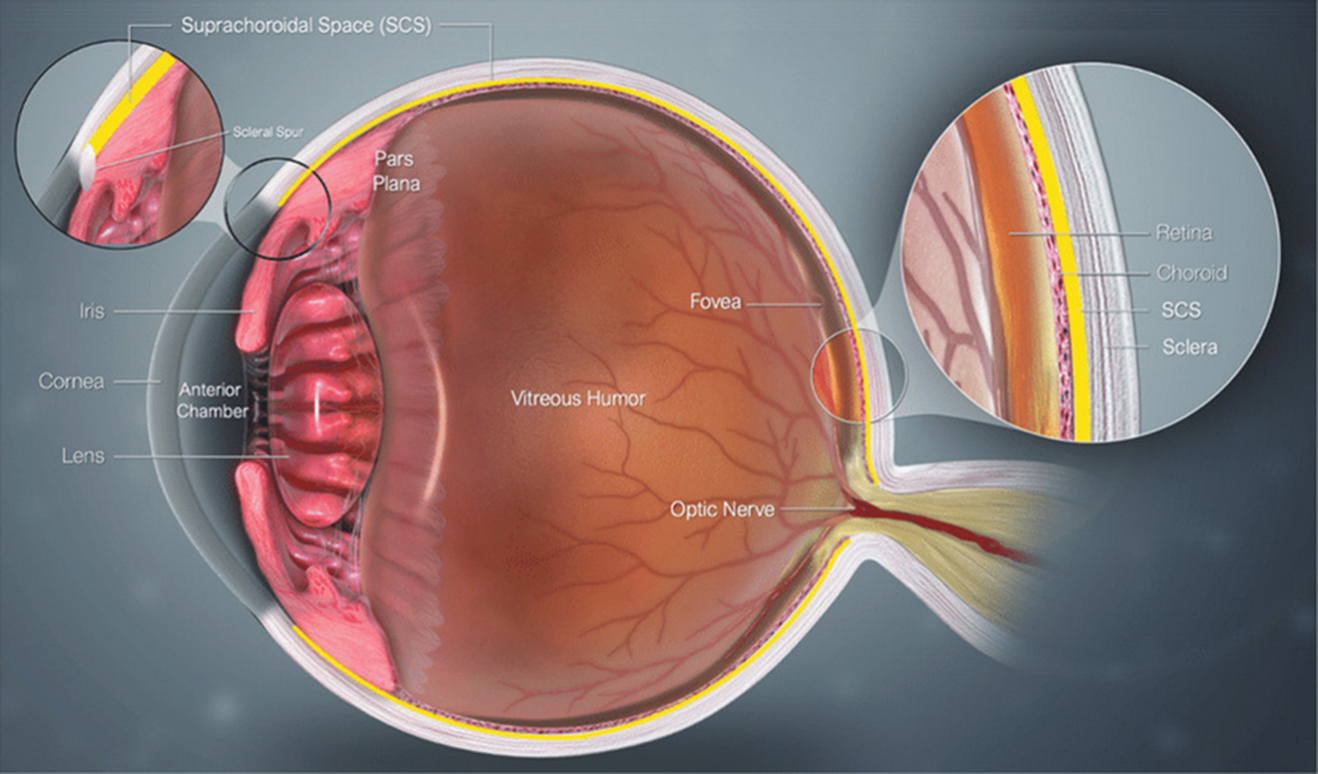
Cross-sectional view of the anterior segment of the eye highlighting the choroid and suprachoroidal space. [Reprinted with permission from Hancock *et al.*, “Biomechanics of suprachoroidal drug delivery: From benchtop to clinical investigation in ocular therapies,” Expert Opin. Drug Delivery **18**, 777–788 (2021). Copyright 2021 Authors, licensed under a Creative Commons Attribution (CC BY) license.]

The TM outflow pathway is referred to as the *conventional adaptive route* (ConvAR) and includes the TM, the Schlemm canal, the collector channels, and the episcleral venous system. The US outflow pathway is referred to as the *unconventional adaptive route* (UncAR) and includes the iris root and the interstitial spaces of the ciliary muscle through which AH flows into the suprachoroidal space (see Fig. 4). The motion of the AH fluid through the anterior segment of the eye is driven by

• the hydraulic pressure difference between PC and AC, AC and episcleral vein, and AC and Sch space;
• the temperature difference between the iris surface (37 °C) and cornea surface (25 °C).

The hydraulic contribution determines the magnitude of AH volumetric flow rate, whereas the thermal contribution controls the AH direction across the AC. Aqueous humor viscosity may be an important factor in AH flow of glaucomatous eyes,^19^ as can also be seen from relations (6) and (7) which show that, for a given pressure drop, the aqueous fluid velocity increases as the fluid becomes more inviscid and decreases if the fluid gets more viscous, as expected from physical intuition. Fluid temperature is responsible for the fact that AH flow is almost tangential to the iris surface, as depicted in Fig. 1. This is numerically confirmed by the 3D finite element simulations by Wang *et al.*^20^ where the mass and momentum balance equations for the fluid are coupled to the energy balance equation for the temperature in which the Boussinesq approximation is adopted to relate fluid density to temperature.

## III. 3D MODEL FOR AQUEOUS HUMOR DYNAMICS

In this section, we formulate the three-dimensional (3D) geometric and differential model to study the processes of AH circulation and drainage in the human eye.

### A. 3D geometric representation

**FIG. 5.**
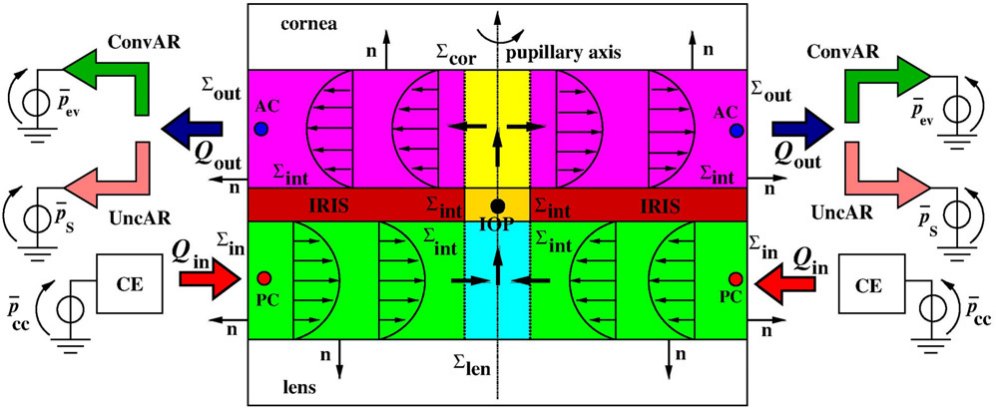
Cross-sectional view of the geometrical scheme of the anterior segment of the eye. The green, gold, brown, and magenta rectangles represent the posterior chamber, the pupil, the iris, and the anterior chamber, respectively. The other symbols and objects and their meaning are described in the main text.

Figure 5 shows a cross-sectional view of the geometrical representation of the anterior segment of the eye considered in this article. The PC and AC are two disks obtained by rotation around the pupillary axis of the green and magenta rectangles, respectively. The pupil is a cylinder obtained by rotation around the pupillary axis of the gold rectangle. The iris is a hollow disk obtained by rotation around the pupillary axis of the brown rectangles, which separates the PC disk from the AC disk. The PC disk is connected to the ciliary capillary pressure ${\bar{p}}_{\text{cc}}$ by the ciliary epithelium (CE) compartment block. The red arrows indicate the volumetric flow rate $Q_{\text{in}}$ that is secreted by the ciliary epithelium and enters the PC. The cyan and yellow rectangles are subsets of the PC and AC, respectively, and represent the transition regions for fluid passage from the PC, through the pupil, into the AC, as indicated by the thick black arrows crossing the vertical dashed lines. The dark blue arrows indicate the AH volumetric flow rate $Q_{\text{out}}$ that is drained out of the AC. This flow rate is divided into two components. The first component of $Q_{\text{out}}$ goes into the ConvAR, which is connected to ground by the episcleral vein pressure ${\bar{p}}_{\text{ev}}$. The second component of $Q_{\text{out}}$ goes into the UncAR, which is connected to ground by the suprachoroidal space pressure ${\bar{p}}_{S}$.

### B. 3D fluid model

Let us denote by $\Omega_{\text{PC}},\;\Omega_{\text{PC},\text{tr}},\;\Omega_{\text{IOP}}$, v $\Omega_{\text{AC},\text{tr}}$, and $\Omega_{\text{AC}}$ the 3D subdomains that are obtained by rotation of the green rectangles; the cyan, gold, and yellow rectangles; and the magenta rectangles in Fig. 5 around the pupillary axis, and let

$$\Omega =\Omega_{\text{PC}}\cup \Omega_{\text{PC},\text{tr}}\cup \Omega_{\text{IOP}}\cup \Omega_{\text{AC},\text{tr}}\cup \Omega_{\text{AC}}.$$

The surface delimiting the boundary of Ω is denoted by Σ, and the outward unit normal vector on Σ is indicated by **n**. The boundary surface Σ is the union of the disjoint boundaries $\Sigma_{\text{in}}$ (interface between the PC and the CE which secretes the AH flowing into the PC), $\Sigma_{\text{len}}$ (interface between the PC and the lens), $\Sigma_{\text{int}}$ (internal surface separating the PC from the iris, the iris from the pupil and the iris from the AC), $\Sigma_{\text{cor}}$ (interface between the AC and the cornea), and $\Sigma_{\text{out}}$ (interface between the AC and the outflow pathway region, comprising the trabecular meshwork and the ciliary muscle).

Throughout the remainder of the article, we make the following assumptions: (a) constant fluid density (water), (b) no net production of fluid inside Ω, (c) stationary flow, (d) small Reynolds number, and (e) no gravitational effects. Under assumptions (a)–(e), the 3D model for the AH fluid is represented by the following differential system for fluid velocity **v** and fluid pressure *p*:

(1)
∇·v=0, (1a)

∇·T=0, (1b)

$$T=-pI+2\mu_{fl}\nabla_{s}v,$$ (1c)

where $\nabla_{s}v=(\nabla v+{\left(\nabla v\right)}^{T})/2$ is the symmetric gradient of **v** (the strain rate, units: $s^{-1}$) and *μ_fl_* is the dynamic viscosity of the fluid (units: Pa s). The system of partial differential equations (PDE) (1) constitutes the conservative form of the Stokes model for a viscous incompressible fluid in stationary motion in the 3D domain Ω under the action of a given volumetric flow rate $\bar{Q}$ and given external pressures ${\bar{p}}_{\text{cc}},\;{\bar{p}}_{\text{ev}}$, and ${\bar{p}}_{s}$. The boundary conditions that complement system (1) read on $\Sigma_{\text{in}}:$

(2)
$$\int_{\Sigma_{\text{in}}}v\cdot n\;d\Sigma =-Q_{\text{in}},$$ (2a)

$$Q_{\text{in}}=Q_{\text{in}}(p_{\text{PC}},{\bar{p}}_{\text{cc}}),$$ (2b)

$$\begin{array}{c} \text{on}\;\Sigma_{\text{len}}\cup \Sigma_{\text{int}}\cup \Sigma_{\text{cor}}:v=0, \end{array}$$ (2c)

$$\begin{array}{c} \text{on}\;\Sigma_{\text{out}}:\int_{\Sigma_{\text{out}}}v\cdot n\;d\Sigma =Q_{\text{out}}, \end{array}$$ (2d)

$$Q_{\text{out}}=Q_{\text{out}}(p_{\text{AC}},{\bar{p}}_{\text{ev}},{\bar{p}}_{S}).$$ (2e)

Equation (2a) expresses the balance between the volumetric flow rate that flows into the PC and the volumetric flow rate that flows out of the PC. Equation (2b) is the functional relation between the trace $p_{\text{PC}}$ of the fluid pressure on $\Sigma_{\text{in}}$ on the side of the PC and the blood pressure ${\bar{p}}_{\text{cc}}$ in the ciliary capillaries. Equation (2c) expresses the no-slip condition for the aqueous fluid at the wall boundaries. Equation (2d) is the functional relation between the trace $p_{\text{AC}}$ of the fluid pressure on $\Sigma_{\text{out}}$ on the side of the AC and the episcleral vein pressure ${\bar{p}}_{\text{ev}}$ and the suprachoroidal space pressure ${\bar{p}}_{S}$.

## IV. REDUCED MODEL FOR AH FLOW

The solution of the 3D continuum model (1) and (2) requires an intensive computational effort because of the coupling between the description of AH motion inside the domain Ω and the description of AH secretion, represented by the functional relation (2b), and AH drainage, represented by the functional relation (2e). As the focus of this work is on the study of the role of conventional and unconventional outflow pathways in the drainage of AH, an alternate approach based on model reduction is desirable, especially in the case where multiple simulation tests have to be performed on different input datasets.

### A. Reduction of momentum balance

The first step to derive the reduced model for AH flow in the anterior segment of the eye is to solve the momentum balance equation (1b) under suitable simplifying assumptions.

**FIG. 6.**
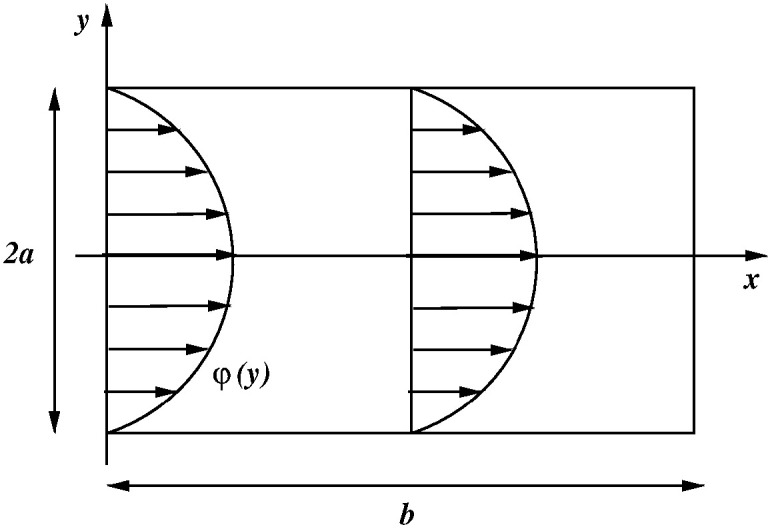
The domain $\Omega_{2D}$ represents any of the green or magenta rectangles in Fig. 5. The length is *b* and the height is 2*a.*

To this purpose, we introduce the two-dimensional domain $\Omega_{2D}$ illustrated in Fig. 6 to represent any of the green or magenta rectangles. We denote by *x*, *y* the spatial coordinates in $\Omega_{2D}$, and $e_{x}$ and $e_{y}$ the unit vectors of the *x* and *y* axes, respectively. Then, we make the following assumptions:

**A.1:** The spatial distributions of fluid velocity and pressure in the 3D domain Ω do not depend on the rotation angle around the pupillary axis.
**A.2:** The fluid velocity vector **v** has the profile of Poiseuille flow along the *x* coordinate, i.e.,
$$v_{y}(x,y)=0\;\forall (x,y)\in \Omega_{2D},$$ (3a)
$$v_{x}(x,y)=\tilde{V}\varphi (y)\;\forall (x,y)\in \Omega_{2D},$$ (3b)
  where $\tilde{V}$ is a constant and $\varphi (y)=\frac{3}{2}(1-{\left(y/a\right)}^{2})$, in such a way that $\tilde{V}$ coincides with the mean integral value of *v_x_* over [−a, a].

Using assumptions **A.1** and **A.2** into the constitutive law (1c) for the fluid stress tensor yields

$$\begin{array}{c} T(x,y)=\left[\begin{array}{cc} 2\mu_{fl}\frac{\partial v_{x}(x,y)}{\partial x}-p(x,y) & \frac{\mu_{fl}}{2}\left(\frac{\partial v_{y}(x,y)}{\partial x}+\frac{\partial v_{x}(x,y)}{\partial y}\right)\\ \frac{\mu_{fl}}{2}\left(\frac{\partial v_{y}(x,y)}{\partial x}+\frac{\partial v_{x}(x,y)}{\partial y}\right) & 2\mu_{fl}\frac{\partial v_{y}(x,y)}{\partial y}-p(x,y) \end{array}\right]\\ =\left[\begin{array}{cc} -p(x,y) & -\frac{3\mu_{fl}}{2}\tilde{V}\frac{y}{a^{2}}\\ -\frac{3\mu_{fl}}{2}\tilde{V}\frac{y}{a^{2}} & -p(x,y) \end{array}\right]. \end{array}$$

Replacing the above expression into (1b) yields

$$\begin{array}{c} \nabla \cdot T(x,y)=\left[\begin{array}{c} -\frac{\partial p(x,y)}{\partial x}-\frac{3\mu_{fl}}{2a^{2}}\tilde{V}\\ -\frac{\partial p(x,y)}{\partial y} \end{array}\right]=\left[\begin{array}{c} 0\\ 0 \end{array}\right]. \end{array}$$ (4)

The second equation in (4) implies that *p* does not depend on *y*, while the first equation in (4) yields

$$\tilde{V}=-\frac{2a^{2}}{3\;\mu_{fl}}\frac{\partial p(x)}{\partial x}.$$ (5)

Since the left-hand side in (5) is a constant, we see that the fluid pressure varies linearly with *x*, and the velocity in the PC and AC is given by the following discrete Darcy's law:

$$\tilde{V}=K_{\Omega_{2D}}\Delta p,$$ (6)

where Δp:=p(0)−p(b) and

$$K_{\Omega_{2D}}=\frac{2a^{2}}{3\;\mu_{fl}b}$$ (7)

is the specific hydraulic conductance of the 2D domain $\Omega_{2D}$ (units: $m\;s^{-1}\;{\text{Pa}}^{-1}$).

### B. Reduction of mass balance

The second step to derive the reduced model for AH flow in the anterior segment of the eye is to solve the mass balance equation (1a) under suitable simplifying assumptions.

**FIG. 7.**
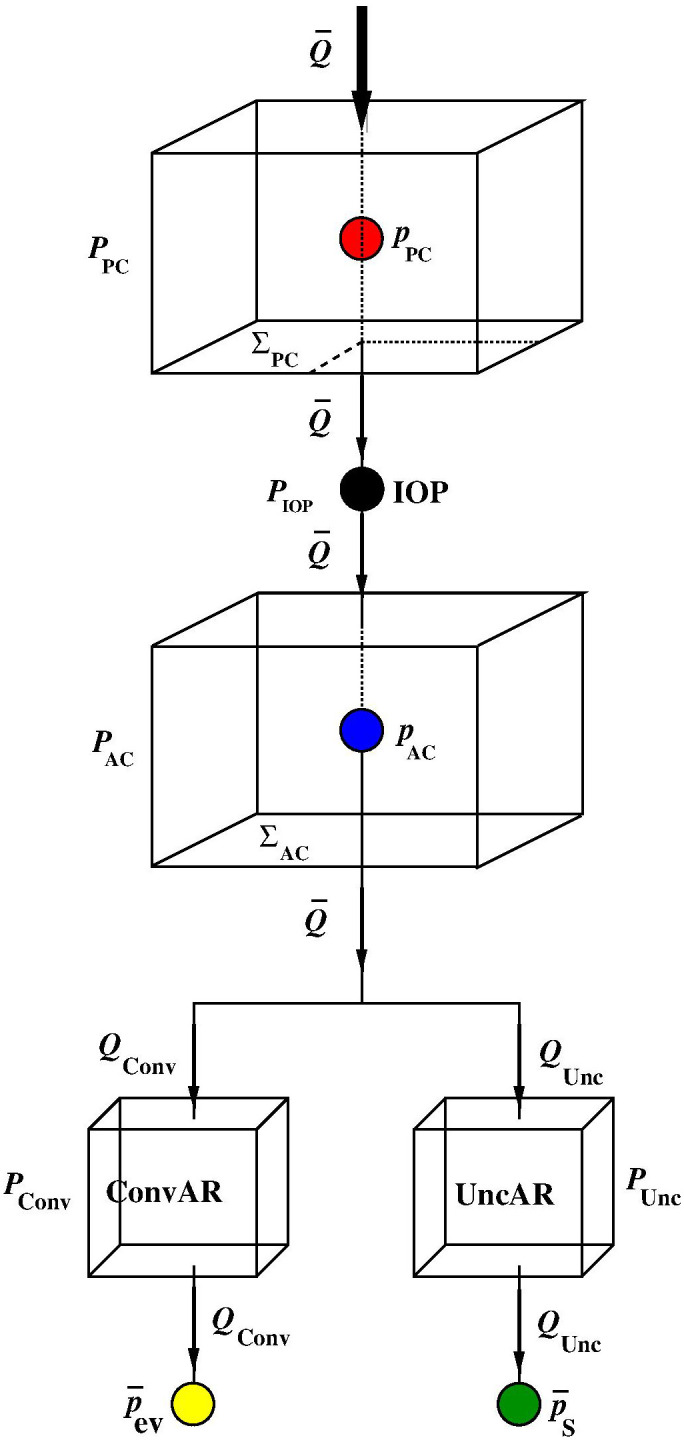
Compartment equivalent representation of the anterior segment of the eye.

To this purpose, we denote by *V* a bounded open domain of ${\mathbb{R}}^{3}$ with sufficiently smooth boundary ∂V and we denote by $n_{\partial V}$ the unit outward normal vector on ∂V. For any differentiable vector field $Z:V\to R^{3}$ (units: Z), we denote by

$$\Phi_{V}(Z)=\int_{V}Z\cdot n_{\partial V}\;d\partial V$$

the net flux of **Z** across the volume boundary ∂V (units: $Z\;m^{2}$). In the case where **Z** is a velocity field (units: $m\;s^{-1}$), the next flux $\Phi_{V}(Z)$ has the physical meaning of volumetric flow rate (units: $m^{3}\;s^{-1}$. Then, we introduce four fluid parallelepipeds, $P_{\text{PC}},\;P_{\text{AC}}$, $P_{\text{Conv}}$, and $P_{\text{Unc}}$, and one node, $P_{\text{IOP}}$, illustrated in Fig. 7 to represent the compartment equivalent scheme of the anterior segment of the eye.

We let $\omega_{\text{PC}}=\Omega_{\text{PC}}\cup \Omega_{\text{PC},\text{tr}}$ and $\omega_{\text{AC}}=\Omega_{\text{AC}}\cup \Omega_{\text{AC},\text{tr}}$, and we denote by $\Omega_{\text{TM}}$ and $\Omega_{\text{cm}}$ the three-dimensional regions of the anterior segment of the eye that are occupied by the TM and the ciliary muscle, respectively. The parallelepipeds and the node enjoy the following properties:

**P.1:**
$$\Phi_{P_{\text{PC}}}(v)=\Phi_{\omega_{\text{PC}}}(v),$$ (8a)
$$\Phi_{P_{\text{AC}}}(v)=\Phi_{\omega_{\text{AC}}}(v),$$ (8b)
$$\Phi_{P_{\text{Conv}}}(v)=\Phi_{\Omega_{\text{TM}}}(v),$$ (8c)
$$\Phi_{P_{\text{Unc}}}(v)=\Phi_{\Omega_{\text{cm}}}(v).$$ (8d)
• **P.2:** the volume of $\Omega_{\text{IOP}}$ is “lumped” into the node $P_{\text{IOP}}$;
• **P.3:** the aqueous fluid flows only through the upper and lower surfaces of each parallelepiped.
  Property **P.1** expresses the fact that each parallelepiped is equivalent to its ocular counterpart from the point of view of mass balance. Property **P.2** expresses the fact that the volume of the pupil is neglected with respect to the volume of the other compartments. Property **P.3** expresses the fact that the aqueous fluid is assumed to flow in the direction connecting the top and bottom surfaces, as represented in Figs. 6 and 7.
  We make the following assumptions:
• **P.4:** The fluid pressure in $P_{\text{PC}}$ is constant and equal to $p_{\text{PC}}$ (indicated by the red bullet in Fig. 7);
• **P.5:** the fluid pressure of node $P_{\text{IOP}}$ is constant and equal to IOP (indicated by the black bullet in Fig. 7);
• **P.6:** the fluid pressure in $P_{\text{AC}}$ is constant and equal to $p_{\text{AC}}$ (indicated by the dark blue bullet in Fig. 7);
**P.7:** the functional relation (2b) is defined as
$$Q_{\text{in}}=\bar{Q},$$ (9)
  where $\bar{Q}$ is a given input parameter representing the volumetric flow rate secreted by the CE
**P.8:** the functional relation (2e) is defined as
$$Q_{\text{out}}=Q_{\text{Conv}}+Q_{\text{Unc}},$$ (10)
  where $Q_{\text{Conv}}$ represents the AH volumetric flow rate drained out through the ConvAR and $Q_{\text{Unc}}$ represents the AH volumetric flow rate drained out through the UncAR. The mathematical expressions of $Q_{\text{Conv}}$ and $Q_{\text{Unc}}$ as a function of the biophysical properties of $P_{\text{Conv}}$ and $P_{\text{Unc}}$ will be provided in Sec. VI.

We now write the mass balance equation (1a) over $\omega_{\text{PC}}$, $\Omega_{\text{IOP}}$, and $\omega_{\text{AC}}$. Using property **P.3**, **P.2**, and **P.7**, in conjuction with the discrete Darcy law (6), we obtain the reduced-order model of AH circulation and drainage

(11)
$$-\bar{Q}+L_{\omega_{\text{PC}}}\left(p_{\text{PC}}-\text{IOP}\right)=0,$$ (11a)

$$-L_{\omega_{\text{PC}}}\left(p_{\text{PC}}-\text{IOP}\right)+L_{\omega_{\text{AC}}}\left(\text{IOP}-p_{\text{AC}}\right)=0,$$ (11b)

$$-L_{\omega_{\text{AC}}}\left(\text{IOP}-p_{\text{AC}}\right)+Q_{\text{Conv}}+Q_{\text{Unc}}=0.$$ (11c)

The quantities $L_{\omega_{\text{PC}}}$ and $L_{\omega_{\text{AC}}}$ are the hydraulic conductances of the PC and AC, respectively (units: $m^{3}\;s^{-1}\;{\text{Pa}}^{-1}$), defined as

$$L_{\omega_{\text{PC}}}=K_{\omega_{\text{PC}}}\Sigma_{\text{PC}},$$ (11d)

$$L_{\omega_{\text{AC}}}=K_{\omega_{\text{AC}}}\Sigma_{\text{AC}}.$$ (11e)

$\Sigma_{\text{PC}}$ and $\Sigma_{\text{AC}}$ being the cross-sectional areas illustrated in Fig. 7. The cross-sectional areas cannot be explicitly characterized as a function of measurable biophysical parameters so that the hydraulic conductances must be determined through a calibration procedure, as described in Sec. VII. The quantities $Q_{\text{Conv}}$ and $Q_{\text{Unc}}$ are the volumetric flow rates of the conventional and unconventional adaptive routes. Their mathematical characterization will be provided in Sec. VI.

## V. THE CONCEPTUAL MODEL

In this section, we translate the reduced model (11) into an equivalent electric circuit. The adoption of this approach in ocular pathophysiology is well known^2^ and has a twofold advantage: (a) it allows us to write AH model equations in terms of Kirchhoff's current law, expressing mass conservation at each node of the circuit, and Ohm's law for any hydraulic conductance in the circuit, expressing the discrete Darcy law (6), and (b) it helps us explicitly characterize $Q_{\text{Conv}}$ and $Q_{\text{Unc}}$ by means of their electric analogs.

**FIG. 8.**
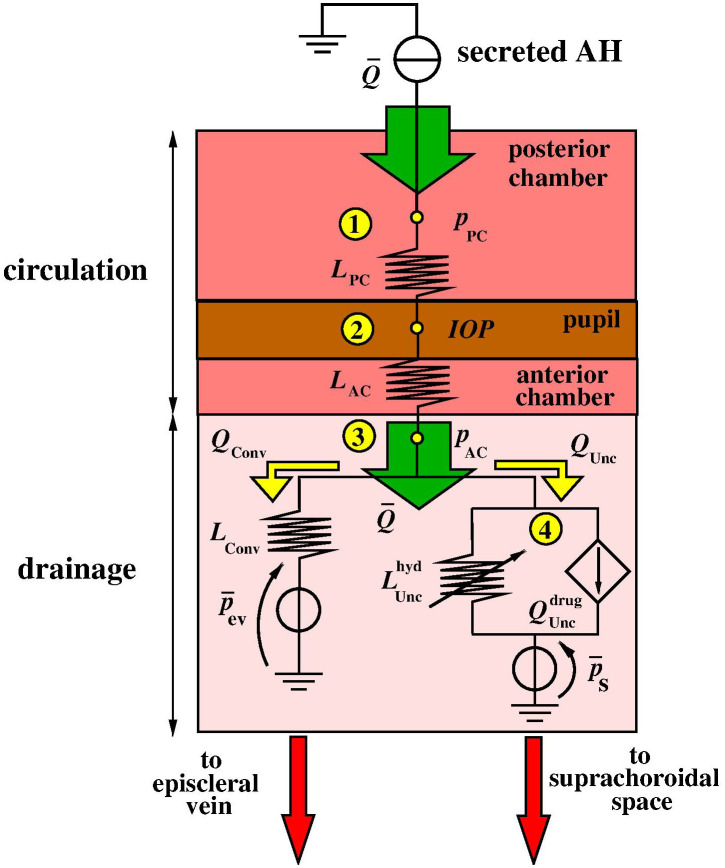
Electric equivalent circuit representing AH circulation between the PC and AC and AH drainage through conventional and unconventional adaptive routes. The current source at the top of the scheme represents the AH volumetric flow rate that is secreted by the ciliary processes. The quantities $L_{\text{PC}}$ and $L_{\text{AC}}$ represent the hydraulic facilities of the PC and AC, respectively. The green arrow, representing the secreted AH volumetric flow rate, divides into two contributions, $Q_{\text{Conv}}$, representing the portion of AH volumetric flow rate that is drained out through the conventional outflow pathway, and $Q_{\text{Unc}}$, representing the portion of AH volumetric flow rate that is drained out through the unconventional outflow pathway.

Figure 8 is a conceptual model of the various processes described in Sec. II and is the translation of the reduced-order model (11) based on the analogy between fluid variables and electric variables described in Sacco *et al.*^9^ according to which electric potential (units: V) is the analog of fluid pressure (units: mm Hg), while electric current (units: A) is the analog of volumetric flow rate (units: $m^{3}s^{-1}$).

The input data of the model are the volumetric flow rate $\bar{Q}$ of AH, represented by the current source at the top of the scheme in Fig. 8, and the voltage sources ${\bar{p}}_{\text{ev}}$ and ${\bar{p}}_{S}$, representing the blood pressure in the episcleral vein (ev) and Sch space, respectively. The current source is the equivalent electric parameter that is used in the present article to describe AH secretion from the ciliary processes of the eye. We refer to Raviola and Raviola^21^ for a description of the cellular and subcellular structure of the ciliary epithelium (CE), Sala *et al.*^22^ for a mathematical model and simulation of transmembrane fluid flow, and Sacco *et al.*^23^ for a mathematical model and simulation of the AH secretion process.

The upper block of the scheme represents the process of AH circulation between the PC and the AC. The symbols $L_{\text{PC}}$ and $L_{\text{AC}}$ represent the hydraulic conductance of the PC and AC, respectively (units: $m^{3}\;s^{-1}\;\text{mm}\;{\text{Hg}}^{-1}$); the quantities $p_{\text{PC}}$ and $p_{\text{AC}}$ represent the pressure exerted by the fluid in the PC and AC, respectively (units: mm Hg), while the quantity *IOP* represents the intraocular pressure resulting from the balance among secretion, circulation, and drainage (units: mm Hg).

The lower block of the scheme represents the process of AH drainage. The quantities $Q_{\text{Conv}}$ and $Q_{\text{Unc}}$ are the volumetric flow rates of AH leaving the eye through the conventional and unconventional pathways, respectively (units: $m^{3}s^{-1}$). The quantity $L_{\text{Conv}}$ is the hydraulic conductance of the TM (units: $m^{3}\;s^{-1}\;\text{mm}\;{\text{Hg}}^{-1}$). The quantity $L_{\text{Unc}}^{\text{hyd}}$ (units: $m^{3}\;s^{-1}\;\text{mm}\;{\text{Hg}}^{-1}$) is the hydraulic conductance representing the adaptive response of the uveoscleral outflow pathway to the increase of TM resistance. The quantity $Q_{\text{unc}}^{\text{drug}}$ (units: $m^{3}s^{-1}$) is the current source representing the adaptive response of the uveoscleral outflow pathway to the administration of an IOP-lowering drug. The hydraulic conductance $L_{\text{Unc}}^{\text{hyd}}$ is a nonlinear function of the pressure difference $p_{\text{AC}}-{\bar{p}}_{S}$, whereas the volumetric flow rate $Q_{\text{Unc}}^{\text{drug}}$ is a nonlinear function of the amount of topical drug that is administered to lower the IOP. The mathematical functional form of $L_{\text{Unc}}^{\text{hyd}}$ and $Q_{\text{Unc}}^{\text{drug}}$ will be specified in Sec. VI.

## VI. MODEL EQUATIONS

The application of Kirchhoff current law (KCL) at nodes 1, 2, 3, and 4 in the electric scheme of Fig. 8 leads to the following system of algebraic equations:

$$-\bar{Q}+Q_{12}=0,$$ (12a)

$$-Q_{12}+Q_{23}=0,$$ (12b)

$$-Q_{23}+Q_{\text{Conv}}+Q_{\text{Unc}}=0,$$ (12c)

$$-Q_{\text{Unc}}+Q_{\text{Unc}}^{\text{hyd}}+Q_{\text{Unc}}^{\text{drug}}=0.$$ (12d)

The quantities *Q*_12_ and *Q*_23_ are the AH volumetric flow rates between nodes 1 and 2 and nodes 2 and 3. The quantities $Q_{\text{Conv}}$ and $Q_{\text{Unc}}$ are the AH volumetric flow rates throughout the conventional and unconventional outflow pathways. The quantity $Q_{\text{Unc}}^{\text{hyd}}$ is the hydraulic-dependent component of the AH volumetric flow rate throughout the unconventional outflow pathway. The quantity $Q_{\text{Unc}}^{\text{drug}}$ is the drug-dependent component of the AH volumetric flow rate throughout the unconventional outflow pathway.

The application of the discrete Darcy law (6) combined with the definition of the hydraulic conductances (11d) and (11e) leads to the following set of constitutive relations:

(13)
$$Q_{12}=L_{\text{PC}}\left(z-x\right),$$ (13a)

$$Q_{23}=L_{\text{AC}}\left(x-y\right),$$ (13b)

$$Q_{\text{Conv}}=L_{\text{Conv}}\left(y-{\bar{p}}_{\text{ev}}\right),$$ (13c)

$$Q_{\text{Unc}}^{\text{hyd}}=L_{\mathrm{Unc}}^{\text{hyd}}(w)w,$$ (13d)

where $z\equiv p_{\text{PC}},\;x\equiv \text{IOP},\;y\equiv p_{\text{AC}}$ and $w\equiv (y-{\bar{p}}_{S})$.

Each equation in system (13) is the electric counterpart of the discrete analog of Darcy's law relating velocity to pressure gradient under the assumption that pressure is a linearly varying function of position. In particular, relations (13a)–(13c) are linear Ohm's laws, whereas relation (13d) is a nonlinear Ohm's law in which the hydraulic conductance $L_{\text{Unc}}^{\text{hyd}}$ nonlinearly depends on the pressure drop $w=y-{\bar{p}}_{S}$.

Replacing relations (13) into (12) yields

$$-\bar{Q}+L_{\text{PC}}\left(z-x\right)=0,$$ (14a)

$$-L_{\text{PC}}\left(z-x\right)+L_{\text{AC}}\left(x-y\right)=0,$$ (14b)

$$-L_{\text{AC}}\left(x-y\right)+L_{\text{Conv}}\left(y-{\bar{p}}_{\text{ev}}\right)+Q_{\text{Unc}}=0,$$ (14c)

$$-Q_{\text{Unc}}+L_{\text{Unc}}^{\text{hyd}}(w)w+Q_{\text{Unc}}^{\text{drug}}(m)=0,$$ (14d)

where *m* (units: g) is the strength of topical drug in a given dosage form (1 drop/day), defined as the drug concentration times the volume of one drop of the drug.

Relations (14) constitute a system of nonlinear algebraic equations for the dependent variables *x*, *y*, *z*, and *w*. The system can be reduced into a single nonlinear algebraic equation for the sole variable *x* by considering *y*, *z*, and *w* as inputs and expressing them as a function of *x*. Replacing Eq. (14a) into (14b) yields

$$y=x-R_{\text{AC}}\bar{Q}=y(x),$$ (15)

where $R_{\text{AC}}=L_{\text{AC}}^{-1}$ is the hydraulic resistance of the AC (units: $\text{mm}\;\text{Hg}\;s\;m^{-3}$). Using now (14c) and (15), we get

$$x={\bar{p}}_{\text{ev}}+\left(R_{\text{Conv}}+R_{\text{AC}}\right)\bar{Q}-R_{\text{Conv}}Q_{\text{Unc}},$$ (16)

where $R_{\text{Conv}}=L_{\text{Conv}}^{-1}$ is the hydraulic resistance of the TM (units: $\text{mm}\;\text{Hg}\;s\;m^{-3}$) and $Q_{\text{Unc}}$ can be computed from (14d).

Relation (16) is a mathematical translation of the conjecture that the UncAR acts as a relief valve to aqueous humor water flow under pathological conditions.^13^ According to the second term on the right-hand side of (16), an increase of TM resistance, $R_{\text{Conv}}$, turns out into an increase of intraocular pressure, which is counterbalanced by the negative pressure drop $-R_{\text{Conv}}Q_{\text{Unc}}$ associated with the UncAR.

**FIG. 9.**
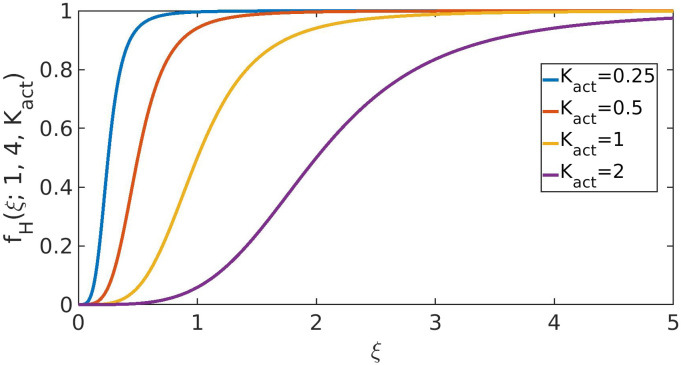
Graph of the Hill function for *β* = 1, in correspondence of several values of $K_{\text{act}}$.

To solve Eq. (16), we need to provide a functional relation for $L_{\text{Unc}}^{\text{hyd}}$ (with respect to the system unknown *w*) and $Q_{\text{Unc}}^{\text{drug}}$ (with respect to the input datum *m*). With this aim, we refer to Keener and Sneyd^24^ and introduce the Hill function associated with the variable ξ≥0,

$$f_{H}(\xi ;\beta ,\;p,\;K_{\text{act}})=\frac{\beta \xi^{p}}{\xi^{p}+K_{\text{act}}^{p}},$$ (17)

where *p* is the Hill coefficient, while *β* and $K_{\text{act}}$ are positive parameters. From now on, in each occurrence, we set *p* = 4. We see that *f_H_* is a sigmoidal monotonically increasing function of *ξ* such that $f(0;\beta ,\;p,\;K_{\text{act}})=0,\;f(K_{\text{act}};\beta ,\;p,\;K_{\text{act}})=\beta /2$ and $f_{H}(+\infty ;\beta ,\;p,\;K_{\text{act}})=\beta$. The rapidity at which *f_H_* reaches the asymptotic value *β* is determined by $K_{\text{act}}$, see Fig. 9 in the case *β*  = 1.

For a generic quantity *U*, we denote by *U_b_* the value of *U* in baseline conditions, and we introduce the relationships

$$w(x)=y(x)-{\bar{p}}_{S}=x-\left(R_{\text{AC}}\bar{Q}+{\bar{p}}_{S}\right),$$ (18a)

$$Z(x)=\text{max}\left\{\frac{w(x)-w_{b}}{w_{b}},0\right\}.$$ (18b)

In the remainder of the text, we assume that the AH volumetric flow rate *Q*, the suprachoroidal pressure ${\bar{p}}_{S}$, and the AC resistance $R_{\text{AC}}$ are set equal to their baseline values $Q_{b},\;{\bar{p}}_{S,b}$, and $R_{\text{AC},b}$, respectively. This assumption implies that

$$Z(x)=\text{max}\left\{\frac{x-x_{b}}{x_{b}-P_{b}},0\right\},$$ (19)

where $P_{b}:={\bar{p}}_{S,b}+R_{\text{AC},b}{\bar{Q}}_{b}$. Based on the numerical values of model parameters (specified in Sec. VII), we have $x_{b}-P_{b}≃3.62\;\text{mm}\;\text{Hg}$. Then, we define

(20)
$$Q_{\text{Unc}}^{\text{hyd}}(x)=L_{\text{unc}}^{\text{hyd}}(Z(x))\left(y(x)-{\bar{p}}_{S}\right),$$ (20a)

$$L_{\text{Unc}}^{\text{hyd}}(Z(x))=L_{\text{Unc},b}\left(1+f_{H}(Z(x);\beta_{\text{unc}}^{\text{hyd}},\;p,\;K_{\text{act},\text{hyd}})\right),$$ (20b)

$$Q_{\text{Unc}}^{\text{drug}}(m)=f_{H}\left(m;\beta_{\text{unc}}^{\text{drug}},\;p,\;K_{\text{act},\text{drug}}\right).$$ (20c)

Let us characterize the values of the parameters $\beta_{\text{max}}$ and $K_{\text{act}}$ for the pressure- and drug-dependent components of the UncAR. In the case of the hydraulic conductance $L_{\text{Unc}}^{\text{hyd}}$, we set

$$K_{\text{act},\text{hyd}}=\frac{p05-{\text{IOP}}_{b}}{{\text{IOP}}_{b}-P_{b}},$$

where $p05=({\text{IOP}}_{b}+{\text{IOP}}_{\text{path}})/2$, ${\text{IOP}}_{\text{path}}=21\;\text{mm}\;\text{Hg}$ being the pathological value of the intraocular pressure, and we evaluate $\beta_{\text{unc},\text{max}}^{\text{hyd}}$ using relation (A4a). The corresponding values of the two parameters are

$$\begin{array}{c} \beta_{\text{unc},\text{max}}^{\text{hyd}}=0.1493,\\ K_{\text{act},\text{hyd}}=0.827. \end{array}$$

In the case of the volumetric flow rate $Q_{\text{Unc}}^{\text{drug}}$, we evaluate $\beta_{\text{unc},\text{max}}^{\text{drug}}$ using relation (B2e). The parameter $K_{\text{act},\text{drug}}$ is given by $0.5M_{\text{tot},\text{abs}}$, where $M_{\text{tot},\text{abs}}$ (units: *μg*) is the total mass of topical drug that is *actually* absorbed by the anterior segment of the eye, given by the following expression:

$$M_{\text{tot},\text{abs}}=Fm_{\text{onedrop}}N_{\text{days}},$$ (21)

where *F* and $m_{\text{onedrop}}$ denote the bioavailability of Latanoprost 0.005% and the mass contained in one drop (units: *μg*), respectively. The corresponding values of the two parameters are

$$\begin{array}{c} \beta_{\text{unc},\text{max}}^{\text{drug}}=7.87\times 10^{-11}\;m^{3}\;s^{-1},\\ K_{\text{act},\text{drug}}=0.547\;\mu g. \end{array}$$

**FIG. 10.**
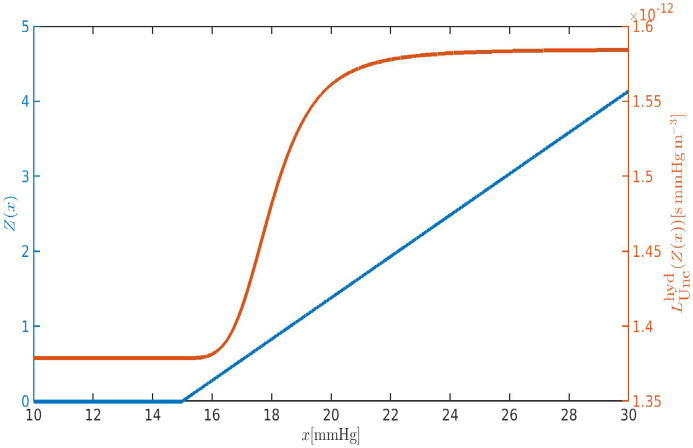
Blue line and blue *y*-axis: plot of *Z* as a function of the intraocular pressure *x* in the interval [10, 30]mmHg. Orange line and orange *y*-axis: plot of $L_{\text{Unc}}^{\text{hyd}}$ as a function of *Z*. The mathematical expressions of *Z* and $L_{\text{Unc}}^{\text{hyd}}$ can be found in Eqs. (19) and (20b), respectively.

The graphs of Z(x) and the corresponding hydraulic conductance $L_{\text{Unc}}^{\text{hyd}}(Z(x))$ as a function of *x* (intraocular pressure) varying between 10 and 30 mm Hg are illustrated in Fig. 10.

Equations (20) are phenomenological relations describing a saturation trend as a function of an increasing parameter. In the case of the pressure-dependent component of the unconventional outflow pathway, the increasing parameter is the deviation from baseline conditions of the pressure difference between anterior chamber and suprachoroidal space, consistent with physiology evidence shown in Refs. 13 and 16. In the case of the drug-dependent component of the unconventional outflow pathway, the increasing parameter is the amount of drug that is administered in a therapy to cure an ocular disease, consistent with the observation that arbitrary increasing drug concentration does not significantly improve the efficacy of the adaptive response of the system. Based on these considerations, the Hill function (17) appears to be a good candidate represent the saturation trend, as it depends on three parameters (the asymptotic value *β*, the activation constant $K_{\text{act}}$, and the Hill exponent *p*) which provide the flexibility required to tune up the sigmoidal shape of the representation.

The constitutive laws (20a) and (20c) are a mathematical translation of the adaptive response of the uveoscleral outflow pathway to an abnormal increase of the TM resistance which is a pathological precondition for the occurrence of glaucoma.^12^ Relation (20a) is a nonlinear Ohm's law representing the hydraulic component of the UncAR, while relation (20c) is a nonlinear drug-controlled current source representing the mechanochemical component of the UncAR.^25^ In the case of (20a), we see that if *w*(*x*) is negative, then $L_{\text{Unc}}^{\text{hyd}}=L_{\text{Unc},b}$, in agreement with the physical consideration that no hydraulic AR is expected if the pressure is below its baseline value. In the case of (20c), we see that arbitrary increasing drug concentration does not significantly improve the efficacy of the mechanochemical AR (saturation effect).

## VII. MODEL CALIBRATION AND PARAMETERS

In this section, we define the values attained by model parameters in baseline conditions, corresponding to a healthy physiological state of the eye of an individual.

Using the data shown by Kiel *et al.*,^3^ the baseline values of IOP (*x_b_*), episcleral vein pressure ( ${\bar{p}}_{\text{ev},b}$), and volumetric flow rate ( ${\bar{Q}}_{b}$) are 15 mm Hg, 8 mm Hg, and $2.74\mu L\;{\text{min}}^{-1}$, respectively (this latter value corresponding to $4.58\times 10^{-11}\;m^{3}\;s^{-1}$). We set the baseline value of PC pressure ( $p_{\text{PC},b}$) equal to 15.5 mm Hg, slightly larger than *x_b_*.

Using the data shown by Ferreira *et al.*,^17^ the baseline value of the AC pressure ( $p_{\text{AC},b}$) is 1950 Pa, corresponding to 14.62 mm Hg, while using the data shown by Emi *et al.*,^16^ the baseline value of the suprachoroidal space pressure ( ${\bar{p}}_{S,b}$) is 4 mm Hg lower than *x_b_* (thus equal to 11 mm Hg).

To determine the baseline values of the hydraulic conductance of PC and AC, $L_{\text{PC},b}$ and $L_{\text{AC},b}$, we use Ohm's law, which gives

$$\begin{array}{c} L_{\text{PC},b}=\frac{{\bar{Q}}_{b}}{p_{\text{PC},b}-x_{b}}=9.17\cdot 10^{-11}\;\text{mm}\;\text{Hg}\;s\;m^{-3},\\ L_{\text{AC},b}=\frac{{\bar{Q}}_{b}}{x_{b}-p_{\text{AC},b}}=1.23\cdot 10^{-10}\;\text{mm}\;\text{Hg}\;s\;m^{-3}. \end{array}$$

To determine the baseline values of the hydraulic conductance of the conventional and unconventional outflow pathways, $L_{\text{Conv},b}$ and $L_{\text{Unc},b}$, we use again Ohm's law starting from the data shown by Kiel *et al.*^3^ of the baseline value of the fraction of AH volumetric flow rate that is absorbed by the conventional pathway, $Q_{\text{Conv},b}=2.45\mu \;L\;{\text{min}}^{-1}$ (corresponding to $4.08\times 10^{-11}\;m^{3}\;s^{-1}$), and the hydraulic component of the unconventional pathway, $Q_{\text{Unc},b}^{\text{hyd}}=0.3\mu \;L\;{\text{min}}^{-1}$ (corresponding to $5\times 10^{-12}\;m^{3}\;s^{-1}$), to obtain

$$\begin{array}{c} L_{\text{Conv},b}=\frac{Q_{\text{Conv},b}}{p_{\text{AC},b}-{\bar{p}}_{\text{ev},b}}=6.16\times 10^{-12}\;\text{mm}\;\text{Hg}\;s\;m^{-3},\\ L_{\text{Unc},b}=\frac{Q_{\text{Unc},b}^{\text{hyd}}}{p_{\text{AC},b}-{\bar{p}}_{S,b}}=1.38\times 10^{-12}\;\text{mm}\;\text{Hg}\;s\;m^{-3}. \end{array}$$

The baseline value of the AH volumetric flow rate of the drug-dependent component of the unconventional pathway, $Q_{\text{Unc},b}^{\text{drug}}$, is equal to zero because we assume that in healthy conditions there is no need to receive a drug treatment.

## VIII. SOLUTION ALGORITHM

Replacing the constitutive laws (13) into Eq. (12d) and substituting the resulting expression for $Q_{\text{unc}}$ into Eq. (16) yields

$$x=T_{m}(x),$$ (22a)

where *T_m_* is the iteration function defined as

$$T_{m}(x)=\frac{\tilde{P}-\tilde{\phi }(m)+P_{b}\phi (x)}{1+\phi (x)}$$ (22b)

having set

$$R_{\text{tot}}:=R_{\text{Conv}}+R_{\text{AC},b},$$ (22c)

$$P_{b}:={\bar{p}}_{S,b}+R_{\text{AC},b}{\bar{Q}}_{b},$$ (22d)

$$\tilde{P}={\bar{p}}_{\text{ev}}+R_{\text{tot}}{\bar{Q}}_{b},$$ (22e)

$$\tilde{\phi }(m)=R_{\text{Conv}}Q_{\text{Unc}}^{\text{drug}}(m),$$ (22f)

$$\phi (x)=R_{\text{Conv}}L_{\text{Unc}}^{\text{hyd}}(Z(x)).$$ (22g)

Referring to Quarteroni *et al.*,^26^ the following definition holds.

**Definition VIII.1.** *Let* G=G(x) *be a given continuous function. The real number ζ is a fixed point of G if*

ζ=G(ζ). (22h)

According to Definition VIII.1, we see that the problem of finding the IOP *x* that solves the nonlinear equation (16) is equivalent to the problem of finding a fixed point of the function *T_m_*. To this purpose, we consider in the remainder of the article the following two situations:

(i) increase of $R_{\text{Conv}}$ from baseline to pathological conditions;
(ii) administration of a topical drug therapy to lower the increase of IOP resulting from (*i*).

The two above situations constitute the modular structure of the Virtual Computational Laboratory (VCL) that we have developed using the Matlab scientific environment.^27^ The mathematical definition and analysis of (*i*) and (*ii*) are addressed in Appendixes A and B, respectively, while their computational performance is investigated in Sec. IX.

## IX. MODEL VALIDATION

In this section, we use the Virtual Computational Laboratory (VCL) proposed in this article to conduct a series of simulations according to the following protocol:

Phase 1: we solve (22a) assuming that
  (a)
$Q_{\text{Unc}}^{\text{drug}}=0$;
  (b) the TM resistance is progressively increased from its baseline value $R_{\text{TM},b}$ to a pathological value $R_{\text{TM},\text{path}}=3R_{\text{TM},b}$.
    The chosen value of $R_{\text{TM},\text{path}}$ is consistent with the observation made in Ref. 28 and with the data of Ref. 18 which show a range between $0.31\;\pm \;0.18\mu L\;{\text{min}}^{-1}\;{\text{mmHg}}^{-1}$ (in human anterior segments treated with 100nM endothelin-1) and $0.49\;\pm \;0.26\mu L\;{\text{min}}^{-1}\;{\text{mmHg}}^{-1}$ (in control human anterior segments.)
Phase 2: we solve (22a) assuming that:
  (a) the TM resistance is kept fixed to $R_{\text{TM},\text{path}}$;
  (b) the mass of an IOP-lowering drug is progressively increased from its baseline value $m_{b}=0\mu g$ to a value $m_{14}=1.094\mu g$, corresponding to a therapy of 14 days with 1 drop/day.

The two-phase protocol has the following clinical interpretation. In Phase 1, the VCL simulates the efficacy of the sole pressure-dependent component of the UncAR to limit the increase of IOP due to a progressive increase of TM resistance, In Phase 2, the VCL simulates the efficacy of the drug-dependent component of the UncAR (in addition to the pressure-dependent component) to lower IOP by the progressive administration of a topical drug (Latanoprost 0.005%) according to a therapy of 14 days with 1 drop/day.

It is worth noting that the simulation of the drug administration therapy does not actually correspond to introducing in the model a “time” variable; rather, time is a parametric variation of the mass of administered drug according to 14 equal increments of 1 drop/day. In the model, the process of drug diffusion across the cornea and throughout the AC is not accounted for; instead, we assume that the quantity of drug increment that is *actually* adsorbed by the eye (i.e., the actual amount that reaches AC) is a percentage of 5% of the administered mass of drug increment. This choice is consistent with the design principles of ocular drug delivery systems.^29^

In the two-phase protocol, we set

$$\begin{array}{c} \beta_{\text{unc}}^{\text{drug}}=\kappa_{\text{drug}}\beta_{\text{unc},\text{max}}^{\text{drug}},\\ \beta_{\text{unc}}^{\text{hyd}}=\kappa_{\text{hyd}}\beta_{\text{unc},\text{max}}^{\text{hyd}}, \end{array}$$

where $\kappa_{\text{drug}}$ and $\kappa_{\text{hyd}}$ are non-negative constants. In the remainder of the text, we set $\kappa_{\text{drug}}=0.33$, in such a way that (*i*) $\beta_{\text{unc}}^{\text{drug}}$ satisfies the sufficient condition (B2f) and (*ii*) the predicted decrease of IOP falls within a range consistent with existing clinical data. Two choices are considered for $\kappa_{\text{hyd}}$, the first is $\kappa_{\text{hyd}}=0$, corresponding to a linear resistor model of the pressure-dependent component of the unconventional adaptive route, and the second is $\kappa_{\text{hyd}}=0.99$, corresponding to a nonlinear resistor model of the pressure-dependent component of the unconventional adaptive route. With both choices, the resulting value of $\beta_{\text{unc}}^{\text{hyd}}$ satisfies the sufficient condition (B3b).

**FIG. 11.**
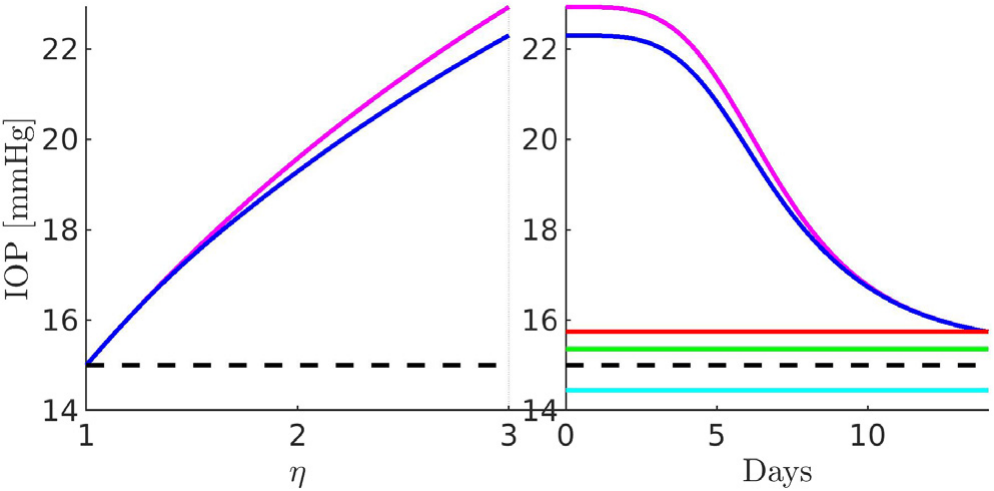
Left panel: IOP as a function of *η*. Right panel: IOP as a function of the number of days of therapy. The red line is characterized by a minimum IOP of 15.73 mmHg, corresponding to a percentage variation of 31.37%. The green line is characterized by a minimum IOP of 15.36 mmHg, corresponding to a percentage variation of 33% (minimum drug efficiency reported in Ref. 30). The cyan line is characterized by a minimum IOP of 14.45 mmHg, corresponding to a percentage variation of 37% (maximum drug efficiency reported in Ref. 30). The values of the parameters for the drug- and pressure-dependent components of the UncAR are $\kappa_{\text{drug}}=0.33$ and $\kappa_{\text{hyd}}=0.99$.

**FIG. 12.**
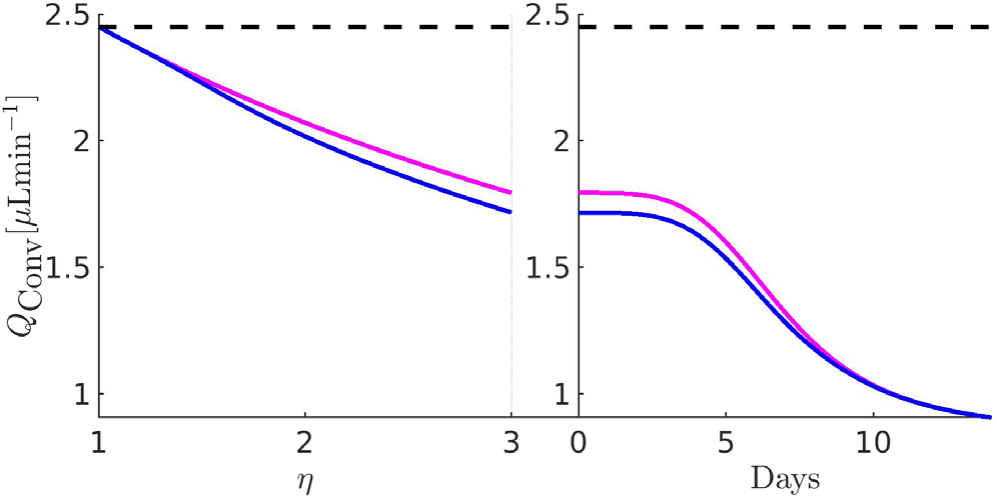
Left panel: $Q_{\text{Conv}}$ as a function of *η*. Right panel: $Q_{\text{Conv}}$ as a function of the number of days of therapy. The values of the parameters for the drug- and pressure-dependent components of the UncAR are $\kappa_{\text{drug}}=0.33$ and $\kappa_{\text{hyd}}=0.99$.

**FIG. 13.**
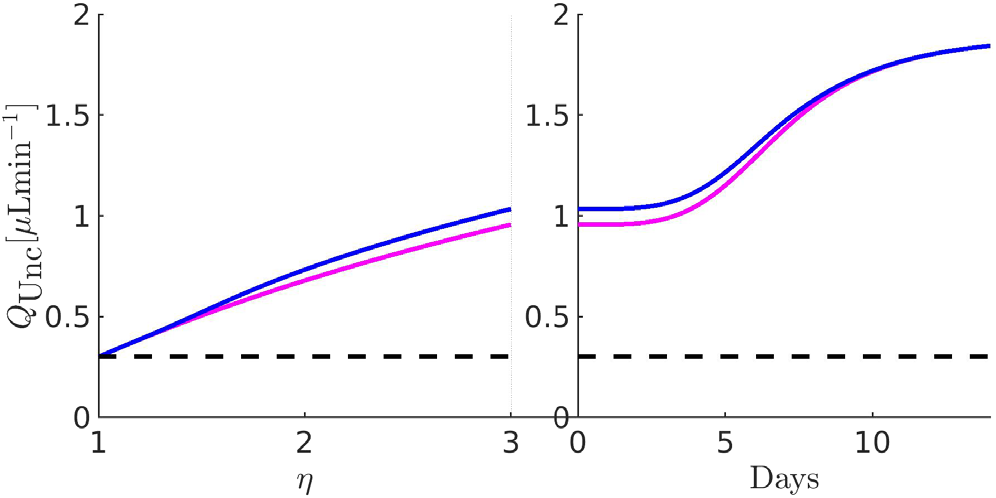
Left panel: $Q_{\text{Unc}}$ as a function of *η*. Right panel: $Q_{\text{Unc}}$ as a function of the number of days of therapy. The values of the parameters for the drug- and pressure-dependent components of the UncAR are $\kappa_{\text{drug}}=0.33$ and $\kappa_{\text{hyd}}=0.99$.

**FIG. 14.**
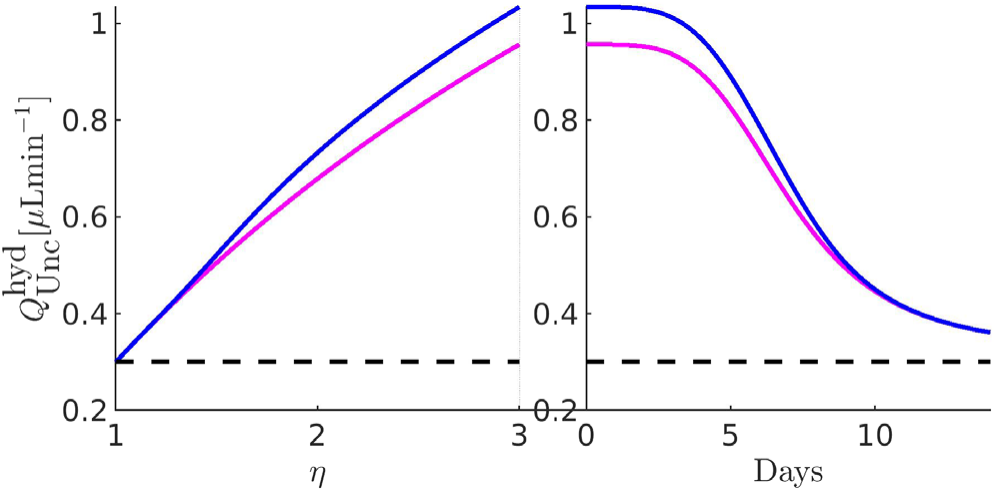
Left panel: $Q_{\text{Unc}}^{\text{hyd}}$ as a function of *η*. Right panel: $Q_{\text{Unc}}^{\text{hyd}}$ as a function of the number of days of therapy. The values of the parameters for the drug- and pressure-dependent components of the UncAR are $\kappa_{\text{drug}}=0.33$ and $\kappa_{\text{hyd}}=0.99$.

**FIG. 15.**
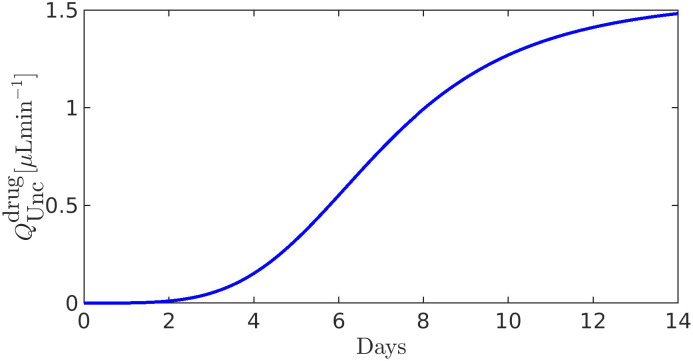
Left panel: $Q_{\text{Unc}}^{\text{drug}}$ as a function of *η*. Right panel: $Q_{\text{Unc}}^{\text{drug}}$ as a function of the number of days of therapy. The values of the parameters for the drug- and pressure-dependent components of the UncAR are $\kappa_{\text{drug}}=0.33$ and $\kappa_{\text{hyd}}=0.99$.

**FIG. 16.**
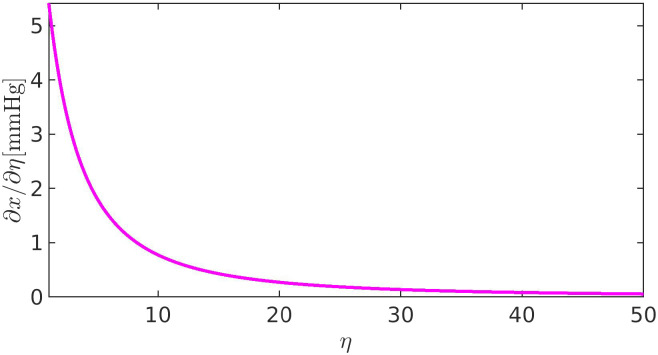
Sensitivity of the IOP with respect to relative variations of $R_{\text{Conv}}$ in the case of a linear resistor model for the hydraulic conductance of the pressure-dependent UncAR.

**FIG. 17.**
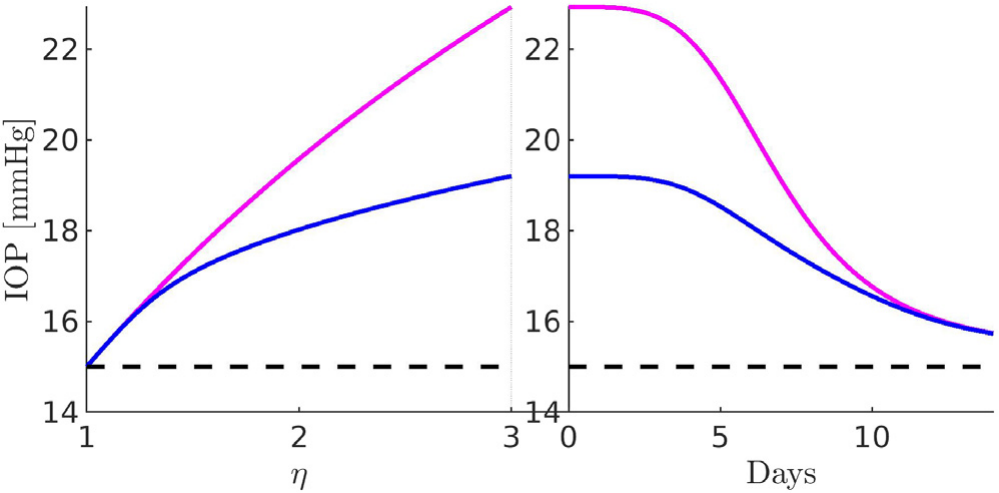
Left panel: IOP as a function of *η*. Right panel: IOP as a function of the number of days of therapy. The values of the parameters for the drug- and pressure-dependent components of the UncAR are $\kappa_{\text{drug}}=0.33$ and $\kappa_{\text{hyd}}=10$.

Figures 11–17 illustrate the results of the two-phase simulation protocol. For each figure, the solid lines in magenta color are obtained with the linear resistor model, the solid lines in blue color are obtained with the nonlinear resistor model, and the dashed lines in black color represent the baseline value of the plotted quantity. The *x*-axis in the left panel represents the ratio $\eta :=R_{\text{Conv}}/R_{\text{Conv},b}$ between the TM resistance and its value at baseline conditions. Thus, a value of 3 on this axis means that the TM resistance is three times the value of the TM resistance at baseline. The *x*-axis in the right panel represents the number of days (14, in total) of the progressive administration of 1 drop/day of Latanoprost 0.005%.

The convergence test of the computational algorithm consisted of terminating the fixed-point iteration (B1a) at the first $k^{*}\geq 0$ such that

$$\frac{\left|x^{\left(k^{*}+1\right)}-x^{\left(k^{*}\right)}\right|}{x^{\left(k^{*}+1\right)}}\leq 10^{-11}.$$

For each simulated condition, $k^{*}$ did not exceed a value of 26. The total elapsed time was on the order of a few seconds, including figure plots, on a laptop equipped with Intel Core i5 processor.

### A. Comments to Fig. 11

The left panel illustrates the intraocular pressure as a function of increasing $R_{\text{Conv}}$ (setting $Q_{\text{drug}}^{\text{hyd}}=0$): we see that both linear and nonlinear models of the hydraulic conductance of the pressure-dependent component of the UncAR predict an increase of IOP as long as the TM resistance becomes higher. The value of the IOP in correspondence of $R_{\text{Conv}}=R_{\text{Conv},\text{path}}$ is almost 23 mm Hg with the linear model and 22.3 mm Hg with the nonlinear model. The right panel illustrates the intraocular pressure as a function of the number of days of therapy (setting $R_{\text{Conv}}=R_{\text{Conv},\text{path}}$): we see that both linear and nonlinear models of the hydraulic conductance of the pressure-dependent component of the UncAR predict a monotonic lowering of the IOP. Until the completion of the first week of therapy, the predicted lowering of IOP is slightly higher with the nonlinear model than with the linear model. In the second week of therapy, both models predict the same monotonic decreasing progression of the IOP, from about 23 to 15.7 mm Hg, corresponding to a percentage reduction of IOP of 31.4% in the case of the linear resistor model and 29.4% in the case of the nonlinear resistor model. These two values of IOP reduction are compatible with the least squares mean percentage IOP changes ranging from 33.3% to 37.3%, published by Eveleth *et al.*^30^ and illustrating a 4-week, dose-ranging study comparing the efficacy of latanoprost 75, 100, and 125 μg/mL to the efficacy of latanoprost 50 μg/mL in the treatment of primary open-angle glaucoma and ocular hypertension. The percentage of IOP reduction of 31.4% (maximum drug efficiency predicted by the model) corresponds to a minimum of IOP equal to 15.73 mm Hg, represented by the horizontal red line. For comparison, the percentage IOP reduction of 33.3% (minimum drug efficiency reported in Ref. 30) corresponds to a minimum of IOP equal to 15.36 mm Hg, represented by the horizontal green line, whereas the percentage IOP reduction of 37.3% (maximum drug efficiency reported in Ref. 30) corresponds to a minimum of IOP equal to 14.45 mm Hg, represented by the horizontal cyan line.

### B. Comments to Fig. 12

The left panel illustrates the conventional AH volumetric flow rate (VFR) as a function of increasing $R_{\text{Conv}}$ (setting $Q_{\text{drug}}^{\text{hyd}}=0$): we see that both linear and nonlinear models of the unconventional hydraulic conductance predict a reduction of $Q_{\text{Conv}}$. In the case of the linear model predicts a decrease of 26.78% of $Q_{\text{Conv}}$ with respect to baseline, whereas the nonlinear model predicts a decrease of 29.95%. The right panel illustrates the conventional AH VFR as a function of the number of days of therapy (setting $R_{\text{Conv}}=R_{\text{Conv},\text{path}}$): we see that both linear and nonlinear models of the hydraulic conductance of the pressure-dependent component of the UncAR predict a monotonic reduction of the conventional AH VFR from about 1.79–0.9 $\mu {\text{Lmin}}^{-1}$, with a predicted maximum reduction of $Q_{\text{Conv}}$ of 49.4%, and with the same trend as in Fig. 11 (right panel).

### C. Comments to Fig. 13

The left panel illustrates the unconventional AH VFR as a function of increasing $R_{\text{Conv}}$ (setting $Q_{\text{drug}}^{\text{hyd}}=0$): in this case, simulation results look complementary to those illustrated in Fig. 12 (left panel). The predicted increase of $Q_{\text{Unc}}$ is larger with the nonlinear model of the unconventional hydraulic conductance than with the linear model, reaching a maximum value of about $1.034\;\mu {\text{Lmin}}^{-1}$ to compare with the value of $0.956\;\mu {\text{Lmin}}^{-1}$ predicted by the linear model. The right panel illustrates the unconventional AH VFR as a function of the number of days of therapy (setting $R_{\text{Conv}}=R_{\text{Conv},\text{path}}$): we see that both linear and nonlinear models of the hydraulic conductance of the pressure-dependent component of the UncAR predict a monotonic increase of the unconventional AH VFR, with a maximum value of $1.843\;\mu {\text{Lmin}}^{-1}$.

### D. Comments to Fig. 14

The left panel coincides with that of Fig. 13. The right panel illustrates the pressure-dependent component of the unconventional AH VFR as a function of the number of days of therapy (setting $R_{\text{Conv}}=R_{\text{Conv},\text{path}}$): we see that both linear and nonlinear models of the unconventional hydraulic conductance predict a monotonic reduction of $Q_{\text{Unc}}^{\text{hyd}}$ until a saturated profile is reached. The reason for the decrease and subsequent saturation of the hydraulic component of the UncAR is that the efficacy in the lowering of IOP is expected to increase with the number of days of therapy, as mathematically expressed by the functional form of $Q_{\text{Unc}}^{\text{drug}}$ in Eq. (20c). Consequently, the increase of $Q_{\text{Unc}}^{\text{drug}}$ has the effect of lowering the pressure in the AC, *y*(*x*), the pressure difference $y(x)-{\bar{p}}_{S,b}$, and, in turn, the pressure-dependent unconventional AH VFR, $Q_{\text{Unc}}^{\text{hyd}}$, which is proportional to $y(x)-{\bar{p}}_{S,b}$ through Eq. (20a).

### E. Comments to Fig. 15

The left panel corresponds to setting $Q_{\text{drug}}^{\text{hyd}}=0$. The right panel illustrates the drug-dependent component of the unconventional AH VFR as a function of the number of days of therapy (setting $R_{\text{Conv}}=R_{\text{Conv},\text{path}}$): this quantity is not an outcome of model computation but a result of post-processing since $Q_{\text{Unc}}^{\text{drug}}$ does not depend on the intraocular pressure *x* [see Eq. (20c)].

### F. Comments to AH mass conservation

In exact arithmetics, the computed value of $Q_{\text{AH}}$ should be *exactly* equal to ${\bar{Q}}_{b}$ so that possible deviations from this value can be regarded as a measure of the accuracy of the model. The maximum absolute difference between ${\bar{Q}}_{b}=2.75\mu {\text{Lmin}}^{-1}$ and $Q_{\text{AH}}$ (computed with the approximate variable *x* obtained by using the nonlinear model of the unconventional hydraulic conductance) is $4.37\times 10^{-11}\mu {\text{Lmin}}^{-1}$, while the same absolute difference is equal to $2.2\times 10^{-15}\mu {\text{Lmin}}^{-1}$ when using the linear model of the unconventional hydraulic conductance. These results demonstrate the high accuracy of the implemented algorithm in enforcing at the numerical level the property of water fluid mass conservation.

## X. DISCUSSION

In this section, we analyze the physiological consistency of the simulation results illustrated in Sec. IX and the clinical relevance of the adaptive routes of the unconventional outflow pathway on the lowering of IOP.

### A. The model for $L_{\text{Unc}}^{\text{hyd}}$ and the lowering of the IOP

The left panel of Fig. 11 suggests that the IOP nonlinearly depends on the TM resistance $R_{\text{Conv}}$ even in the case where a linear resistor electric analog is used for the pressure-dependent component of the unconventional outflow pathway (corresponding to $\kappa_{\text{hyd}}=0$). To understand the cause of this behavior, we study the sensitivity $S_{x}=S_{x}(\eta )$ of IOP (*x*) with respect to a relative change *η* in the TM resistance. We have

$$S_{x}(\eta ):=\frac{\partial x}{\partial \eta }=R_{\text{Conv},b}\left[\frac{{\bar{Q}}_{b}}{1+{\bar{\phi }}_{b}}+L_{\text{Unc},b}\frac{\left(P_{b}-\tilde{P}\right)}{{\left(1+{\bar{\phi }}_{b}\right)}^{2}}\right],$$ (23)

where ${\bar{\phi }}_{b}$ denotes the baseline value of ϕ(x). The plot of $S_{x}(\eta )$ in Fig. 16 reveals that

(1) the IOP continuously increases since $S_{x}(\eta )>0$;
(2) the IOP increase becomes progressively smaller since $S_{x}(\eta )$ is monotonically decreasing;
(3) saturation of the IOP occurs only for unphysiologically elevated values of $R_{\text{Conv}}$.

The above considerations support the conjecture that a linear model for the pressure-dependent adaptive route of the unconventional outflow pathway is not adequate to account for a significant lowering of the IOP as an adaptive response to a pathological increase of $R_{\text{Conv}}$. To overcome this inadequacy, we, therefore, assume that $\kappa_{\text{hyd}}>0$, in such a way that the hydraulic conductance $L_{\text{Unc},b}^{\text{hyd}}$ depends (nonlinearly) on the pressure drop $p_{\text{AC}}-{\bar{p}}_{S}$, consistent with the conjecture of Costagliola *et al.*^13^

The physiological motivation of the nonlinear model (20b) is based on the conjecture formulated by Liton and Gonzalez^11^ of an adaptive regulatory mechanism in the cells of the outflow pathway capable to respond to the mechanical stress originated by changes in the IOP by triggering signals aimed at modulating the flow of AH. The sigmoidal shape of the Hill function (17) is an attempt to include a saturation of the IOP for elevated values of the TM resistance, in agreement with the concept of intraocular pressure homeostasis of keeping ocular pressure within relatively narrow acceptable bounds elaborated by Acott *et al.*^31^

To test the efficacy of the nonlinear model (20b), we illustrate in Fig. 17 the model prediction of IOP as a function of *η* in the case where $\kappa_{\text{hyd}}=10$, corresponding to a value of $\beta_{\text{unc}}^{\text{hyd}}$ that does not satisfy the sufficient condition (B3b). The solid line (blue color) in the left panel, obtained with the nonlinear model (20b), predicts a tendency to saturation of IOP to a value of about 19.2 mm Hg, to be compared with the maximum value of about 23 mm Hg predicted by the linear model. Since the usual criterion for the onset of glaucoma is an IOP higher than 21 mm Hg,^32^ simulation predictions suggest that the nonlinear model for $L_{\text{Unc}}^{\text{hyd}}$ is consistent with an adaptive response of the unconventional outflow pathway capable of restoring IOP homeostasis in the anterior segment of the eye.^31^

### B. The model for $Q_{\text{Unc}}^{\text{drug}}$ and the lowering of the IOP

The effect of drug administration on the uveoscleral outflow is subject to intensive investigation. Pilocarpine causes contraction of the ciliary muscle fibers and compression of extracellular space, thus reducing the uveoscleral outflow.

Atropine, instead, causes relaxation of the ciliary muscle fibers and expansion of the extracellular space, thus increasing the uveoscleral outflow as discussed by Bill^33^ and Johnson *et al.*^15^ Prostaglandin analogs, such as Latanoprost 0.005%, also contribute to lowering of IOP, as resulting from immunohistochemical data that show how the administration of topical prostaglandin F2-*α* is associated with a reduction of collagens within the extracellular matrix between ciliary muscle bundles, thus reducing the outflow resistance and increasing uveoscleral outflow.^34–37^ The experimental measurement of outflow through the unconventional pathway is quite a challenging task because of the difficulty in measuring the flow rate of AH as pointed out by Johnson *et al.*^15^ (Sec. IV). This difficulty provides a motivation to devise alternate methods (indirect techniques) to gain information on the unconventional pathway outflow. In this perspective, the expression (20c) proposed in the present work can be regarded as an original instance of an indirect technique to model the AH volumetric flow rate across the UncAR, secondary to IOP-lowering drug administration.

In the simulations, we set *F* = 0.05 (corresponding to an absorbed mass equal to 5% of the administered mass) and $m_{\text{one}\;\text{drop}}=1.5625\mu g$. Model predictions in the right panels of Figs. 11 and 17 indicate that the reduction of IOP from the value corresponding to pathological conditions is equal to 7.19 mm Hg in the case of the linear resistor model, while the reduction in the case of the nonlinear resistor model is equal to 6.56 mm Hg if $\kappa_{\text{hyd}}=0.99$ and 3.48 mm Hg if $\kappa_{\text{hyd}}=10$. The predicted IOP reduction in the case $\kappa_{\text{hyd}}=10$ is compatible with the data reported by Wang *et al.*^38^ where a mean reduction of 6.56 mm Hg was measured in glaucomatous patients treated with Latanoprost 0.005% for 4 weeks.

## XI. CONCLUSIONS

The adoption of mathematical modeling and computational techniques to advance knowledge in ophthalmology is relatively recent but continuously increasing, as witnessed by the foundation of peer-reviewed journals specifically devoted to the topic (cf. e.g., the website^39^) and the development of editorial initiatives targeted to provide a unified framework to anatomy, physiology, and imaging.^40^

In this article, we propose and analyze a novel mechanism-driven mathematical model of aqueous humor dynamics in the human eye. The formulation is a compartment representation of the anterior segment of the eye based on the analogy between electric and fluid variables illustrated in Sacco *et al.*^9^ The scheme is implemented in a Virtual Computational Laboratory that is used to investigate the adaptive routes exerted by the two components of AH drainage, the conventional and unconventional outflow pathways, to lower an elevated value of intraocular pressure in the eye due to an abnormal increase of the resistance of the TM as proposed by Costagliola *et al.*^13^

The novelty of our model is the mathematical characterization of the unconventional outflow pathway by means of phenomenological constitutive laws that attempt to take into account the principal conjectures raised about the adaptive mechanisms that are put in action by the ciliary muscles to contrast an abnormal increase of the IOP. The proposed model of the UncAR is demonstrated:

• to provide a correct physiological picture of the hydrodynamical conditions in the anterior segment of the eye;
• to reproduce clinical observations in the cure of the eyes of patients affected by glaucoma.

The computational performance of the formulation is corroborated by an *a priori* theoretical analysis of the well-posedness of the fixed-point iteration which is used to numerically solve the nonlinear model equations. The importance of this analysis is that it provides quantitative information about the values of specific model parameters that are seen to play a relevant role in the function of the simulated system.

The proposed mathematical framework is affected by several limitations; specifically:

(1) the electro-osmotic-oncotic pressure gradient which acts at the cellular and subcellular level to drive the fluid from the ciliary capillaries of the ciliary body into the PC is not accounted for in the description of the process of AH secretion;
(2) the outflow pathway routes are described by means of phenomenological laws so that adaptive mechanisms occurring at the cellular scale level in both TM and ciliary muscle are not accounted for;
(3) the processes of drug absorption across the cornea and drug diffusion into the AC of the eye are not included.

Regardless of the above limitations, the model proposed in this article has been demonstrated to be a useful tool to investigate the processes of circulation and drainage of aqueous humor in the anterior segment of the human eye. The obtained predictions of the intraocular pressure and AH flow are in agreement with ocular physiology and clinical data and provide an encouraging basis toward combining the proposed model with the modern techniques of Machine Learning and Artificial Intelligence proposed by Guidoboni *et al.*^41^ and that promise to represent the winning approach to a routine implementation of “personalized medicine” in the cure of ocular diseases.^42^

## XII. FIGURES WITH SIMULATION RESULTS

In this section, we collect the figures illustrating the simulation results described in Sec. IX and discussed in Sec. X.

## Data Availability

The data that support the findings of this study are available within the article.

## APPENDIX A: SUBPROBLEM 1: FROM BASELINE TO PATHOLOGICAL CONDITIONS

The aim of this section is to mathematically describe the response of the pressure-dependent component of the UncAR to a progressive increase of trabecular resistance from its value $R_{\text{Conv},b}$ in baseline conditions to a pathological value $R_{\text{Conv},\text{path}}>R_{\text{Conv},b}$, possibly due to trabecular degeneration secondary to chronic oxidative stress and cellular senescence as conjectured by Cela *et al.* in Ref. 43. In this section, we assume that no drug is administered, i.e., we set *m* = 0 in (22f), and we introduce the following fixed-point iteration to compute an approximation of *x*:

Given $x^{\left(0\right)}$, for every k≥0 until convergence, compute

$$x^{\left(k+1\right)}=T_{0}(x^{\left(k\right)}),$$
(A1a)

$$T_{0}(x)=\frac{\tilde{P}+P_{b}\phi (x)}{1+\phi (x)}.$$
(A1b)

Let us examine the theoretical properties of the fixed-point iteration (A1a). The first question we need to address is if *T*_0_ admits a fixed point $x^{*}$ (existence). The second question is if $x^{*}$ is unique. The third question is if

$$\underset{k\to \infty }{\text{lim}}x^{\left(k\right)}=x^{*}\;(\text{convergence}).$$
(A1c)

### 1. Existence

Let $I_{x}:=[0,+\infty )$. It is immediate to see that for all $x\in I_{x}$, we have

$$R_{\mathrm{Conv}}L_{\text{Unc},b}\leq \phi (x)\leq R_{\text{Conv}}L_{\text{Unc},b}\left(1+\beta_{\text{unc}}^{\text{hyd}}\right).$$
(A2a)

Using (A2a) into (A1b), we obtain the range of *T*_0_

$$A\leq T_{0}(x)\leq B\;\forall x\in I_{x},$$
(A2b)

where

$$A=\frac{\tilde{P}+P_{b}R_{\text{Conv}}L_{\text{Unc},b}}{1+R_{\text{Conv}}L_{\text{Unc},b}\left(1+\beta_{\text{unc}}^{\text{hyd}}\right)},$$
(A2c)

$$B=\frac{\tilde{P}+P_{b}R_{\text{Conv}}L_{\text{Unc},b}\left(1+\beta_{\text{unc}}^{\text{hyd}}\right)}{1+R_{\text{Conv}}L_{\text{Unc},b}}.$$
(A2d)

Since 0<A<B<+∞, the range of *T*_0_ is included in *I_x_*. Then, Theorem 6.1 in Quarteroni *et al.*^26^ ensures that *T*_0_ admits (at least) a fixed point $x^{*}\in I_{x}$.

### 2. Uniqueness and convergence

The following result is a consequence of Theorem 6.1 in Quarteroni *et al.*^26^

**Lemma XIII.1.** *Assume that the following conditions are satisfied:*

**C.1** $T_{0}\in C^{1}(I_{x})$;
**C.2** *there exists a constant* 0<M<1 *such that*
  $$|T_{0}^{\prime }(x)|\leq M\;\forall x\in I_{x}.$$
  (A3a)

*Then, T_0_ has a unique fixed point* $x^{*}\in I_{x}$ *and the sequence* $\left\{x^{\left(k\right)}\right\},\;k\geq 0$*, generated by the fixed-point iteration* (A1a) *converges to* $x^{*}$ *for every choice of* $x^{\left(0\right)}$ *in I_x_.*

Condition **C.1** is satisfied except at *x* = *x_b_*, where $T_{0}^{\prime }$ has a finite jump. This mathematical difficulty, however, is only apparent because if $x\leq x_{b}$, then $\phi (x)=R_{\text{Conv}}L_{\text{Unc},b}^{\text{hyd}}$ so that $T_{0}^{\prime }(x)=0$ (for $x\leq x_{b}$) and (A1a) converges in one iteration. Therefore, we can safely assume $x>x_{b}$. Let

$$\beta_{\text{unc},\text{max}}^{\text{hyd}}:=\frac{\left|x_{b}-P_{b}\right|}{\left|P_{b}-\tilde{P}\right|}\frac{1}{pK_{\text{act},\text{hyd}}^{p}R_{\text{Conv}}L_{\text{Unc},b}}.$$
(A4a)

Assume that

$$\beta_{\text{unc}}^{\text{hyd}}<\beta_{\text{unc},\text{max}}^{\text{hyd}}.$$
(A4b)

Define

$$M=\frac{\left|P_{b}-\tilde{P}\right|}{\left|x_{b}-P_{b}\right|}p\beta_{\text{unc}}^{\text{hyd}}K_{\text{act},\text{hyd}}^{p}R_{\text{Conv}}L_{\text{Unc},b}.$$
(A4c)

We obviously have M<1.

**Theorem XIII.1 (Uniqueness and convergence)**. *Since the* assumptions C.1 *and* ***C.2*** *are satisfied, T_0_ admits a unique fixed point* $x^{*}\in I_{x}$ *and the fixed-point iteration* *(A1a)* *converges to* $x^{*}$ *for every choice of* $x^{\left(0\right)}\in I_{x}$.

## APPENDIX B: SUBPROBLEM 2: ADMINISTRATION OF A TOPICAL DRUG THERAPY

The aim of this section is to mathematically describe the effect of the administration of a topical drug to lower the IOP when the trabecular resistance is characterized by a pathological value. In what follows, we set m≥0 and $R_{\text{Conv}}=R_{\text{Conv},\text{path}}>R_{\text{Conv},b}$, and we use the notation $U_{\text{path}}$ to indicate that the variable *U* (depending on $R_{\text{Conv}}$) is evaluated with $R_{\text{Conv}}=R_{\text{Conv},\text{path}}$. The following fixed-point iteration can be used to compute an approximation of *x*:

Given $x^{\left(0\right)}$, for every k≥0 until convergence, compute

$$x^{\left(k+1\right)}=T_{m,\text{path}}(x^{\left(k\right)}),$$
(B1a)

$$T_{m,\text{path}}(x)=\frac{{\tilde{P}}_{\text{path}}-\tilde{\phi }(m)+P_{b}\phi_{\text{path}}(x)}{1+\phi_{\text{path}}(x)}.$$
(B1b)

Let us examine the theoretical properties of the fixed-point iteration (B1a).

### 1. Existence

We see that for all m≥0,

$$0\leq \tilde{\phi }(m)\leq R_{\text{Conv},\text{path}}\beta_{\text{unc}}^{\text{drug}}.$$
(B2a)

Using (B2a) into (B1b), we obtain the range of $T_{m,\text{path}},$

$$\tilde{A}\leq T_{m,\text{path}}(x)\leq \tilde{B}\;\forall x\in I_{x},$$
(B2b)

where

$$\tilde{A}=\frac{{\tilde{P}}_{\text{path}}-R_{\text{Conv},\text{path}}\beta_{\text{unc}}^{\text{drug}}+P_{b}R_{\text{Conv},\text{path}}L_{\text{Unc},b}}{1+R_{\text{Conv},\text{path}}L_{\text{Unc},b}\left(1+\beta_{\text{unc}}^{\text{hyd}}\right)},$$
(B2c)

$$\tilde{B}=\frac{{\tilde{P}}_{\text{path}}+P_{b}R_{\text{Conv},\text{path}}L_{\text{Unc},b}\left(1+\beta_{\text{unc}}^{\text{hyd}}\right)}{1+R_{\text{Conv},\text{path}}L_{\text{Unc},b}}.$$
(B2d)

Let

$$\beta_{\text{unc},\text{max}}^{\text{drug}}:={\tilde{P}}_{\text{path}}L_{\text{Conv},\text{path}}+P_{b}L_{\text{unc},b},$$
(B2e)

where $L_{\text{Conv},\text{path}}:=R_{\text{Conv},\text{path}}^{-1}$ is the hydraulic conductance of the TM in pathological conditions. Proceeding as in Appendix A 1, we see that if

$$\beta_{\text{unc}}^{\text{drug}}\leq \beta_{\text{unc},\text{max}}^{\text{drug}}$$
(B2f)

then, $T_{m,\text{path}}$ admits (at least) a fixed point $x_{\text{path}}^{*}\in I_{x}$.

### 2. Uniqueness and convergence

Let

$$\beta_{\text{unc},\text{path},\text{max}}^{\text{hyd}}:=\frac{\left|x_{b}-P_{b}\right|}{\left|P_{b}-{\tilde{P}}_{\text{path}}\right|}\frac{1}{pK_{\text{act},\text{hyd}}^{p}R_{\text{Conv},\text{path}}L_{\text{Unc},b}}.$$
(B3a)

Noting that $\tilde{\phi }$ does not depend on *x* so that $d\tilde{\phi }/dx=0$, we can proceed as in Appendix A 2 to conclude that if

$$\beta_{\text{unc}}^{\text{hyd}}<\beta_{\text{unc},\text{path},\text{max}}^{\text{hyd}}$$
(B3b)

then, $T_{m,\text{path}}$ admits a unique fixed point $x_{\text{path}}^{*}\in I_{x}$ and the fixed-point iteration (B1a) converges to $x_{\text{path}}^{*}$ for all $x^{\left(0\right)}\in I_{x}$. As physically expected, we see that the (sufficient) condition on $\beta_{\text{unc}}^{\text{hyd}}$ to be satisfied to ensure the existence of a fixed point is more stringent when the TM undergoes pathological conditions than in normal situations since $\beta_{\text{unc},\text{path},\text{max}}^{\text{hyd}}\leq \beta_{\text{unc},\text{max}}^{\text{hyd}}$.
